# Oral microbiome contribution in colorectal carcinogenesis: polymicrobial interactions and virulence determinants

**DOI:** 10.3389/fcimb.2026.1887826

**Published:** 2026-07-28

**Authors:** Evgeniya Glazunova, Lidiia Astakhova, Alexander Kurnosov, Yuriy Doludin, Alexey Lyundup, Andrey Kostin, Valentin Makarov, Sergey Yudin, Olga Zlobovskaya

**Affiliations:** 1 Federal State Budgetary Institution «Centre for Strategic Planning and Management of Biomedical Health Risks» of the Federal Medical Biological Agency, Moscow, Russia; 2 Emanuel Institute of Biochemical Physics of Russian Academy of Sciences, Moscow, Russia; 3 Coordinating Center for Fundamental Research, National Medical Research Center for Therapy and Preventive Medicine, Moscow, Russia; 4 Educational Resource Center for Cellular Technologies, Peoples' Friendship University of Russia (RUDN), Moscow, Russia

**Keywords:** virulence factors, colorectal cancer, human oral pathobionts, bridge species, CRC diagnosis, biomarkers, polymicrobial Interactions, biofilm

## Abstract

The contribution of the microbiome to sporadic colorectal cancer (CRC) has traditionally been interpreted through the detection of individual tumor-enriched taxa or isolated virulence factors. However, accumulating evidence indicates that CRC-associated microorganisms often act as structured polymicrobial consortia rather than as independent agents. This review proposes an expanded ecological framework for understanding the role of oral taxa in colorectal carcinogenesis. Oral bacteria, including genera *Fusobacterium*, *Parvimonas*, *Peptostreptococcus*, *Porphyromonas*, *Prevotella*, *Streptococcus*, and related taxa, may translocate to the colorectal niche through enteral or hematogenous routes and establish tumor-associated communities that partially recapitulate the structural organization and succession patterns of oral biofilms. Within these communities, *Fusobacterium nucleatum* serves as the primary bridging organism, facilitating interspecies adhesion and spatial organization. *Parvimonas micra*, *Porphyromonas gingivalis*, and *Candida albicans* display potential bridging structural and functional properties, with their roles in cooperative pathogenicity and tumor progression supported by substantial experimental evidence. We integrate three complementary conceptual frameworks: oral microbial complexes (gut co-abundance groups), bridging organisms and the extended driver-passenger model of CRC. This synthesis supports a shift from taxonomy-centered interpretation toward a functional virulome-based view, in which the role of a taxon is defined by its ecological position, interaction network, and virulence determinants. Key oral virulence factors contribute to biofilm formation, epithelial adhesion and invasion, barrier disruption, immune evasion, inflammatory modulation, metabolic remodeling of the tumor microenvironment, and metastatic potential. Because the composition of translocated oral consortia changes gradually within the colorectal niche, successive consortium members and their associated virulence determinants may serve as indirect microbial signatures of CRC progression. Recognizing CRC-associated oral communities as structured, interactive ecosystems has important diagnostic and therapeutic implications. Simultaneous detection of pathobionts, their cooperative clusters, and functionally relevant virulence determinants may improve microbiome-based risk stratification. Moreover, targeted disruption of bridging functions, interspecies interactions, or virulence factor activity may offer a precision strategy to weaken carcinogenic consortia without broad, dysbiosis-promoting antimicrobial pressure.

## Introduction

Early investigations into the association of pathogenic and opportunistic members of the human microbiome with sporadic colorectal cancer (CRC), as well as their potential effects on CRC pathogenesis, relied primarily on the characterization of individual taxa (Dejea et al., 2013; Mu et al., 2020) and, subsequently, of isolated virulence factors (Kaplan et al., 2009; Allen-Vercoe and Jobin, 2014; Guo et al., 2017). This individual-component strategy offers clear advantages for the primary establishment of cause-and-effect relationships (Scott et al., 2019). Studying a pathogen and its virulence factors individually (outside the community context) provides baseline evidence of the mechanisms underlying host damage at the molecular and cellular levels (Scott et al., 2019; Lopès et al., 2020), but it excludes indirect pathways and synergistic effects of pathogenesis. For this reason, current research has shifted its focus toward microbial communities and consortium-level pathogenic potential, and further, toward the ecological niche as a whole, whose complex interaction networks encompass metabolic, inflammatory, and immunological pathways (Kuboniwa et al., 2017; El-Awady et al., 2019; Morse et al., 2019; Horiuchi et al., 2020; Bautista et al., 2026). This paradigm transition from the effects of individual species to the coordinated activity of polymicrobial consortia is grounded in the recognition that most microbiome-associated infectious and chronic diseases arise not from the autonomous output of a single organism, but from the collective impact of a polymicrobial bacterial community (Hajishengallis et al., 2011; Barbosa et al., 2015; Kuboniwa et al., 2017; El-Awady et al., 2019; Morse et al., 2019; Neilands et al., 2019; Horiuchi et al., 2020; Avril and DePaolo, 2021; Périchon et al., 2022; Higashi et al., 2023). Similarly, the focus has moved from individual virulence factors toward a framework that considers the synergistic contribution of virulence determinants within structured microbial communities (Meuric et al., 2013; Barbosa et al., 2015; Higashi et al., 2023; Wronowska et al., 2025). In CRC, microbial colonization of tumor tissue is increasingly viewed as a consortium-level process, driven not by individual taxa alone but by the coordinated activity of microbial communities and the synergistic action of their virulence determinants (Hajishengallis et al., 2011; Dejea et al., 2018; Morse et al., 2019; Tomkovich et al., 2019; Avril and DePaolo, 2021; Kvich et al., 2024; Bautista et al., 2026).

Among taxa with the strongest evidence for CRC association, a substantial proportion are typical oral bacteria. In their native niche, oral communities provide colonization resistance (Higashi et al., 2023), modulate innate and adaptive mucosal immunity (Chen et al., 2022b), maintain barrier integrity (Chen et al., 2022b) and immune homeostasis (Zhang et al., 2008; Warren et al., 2013; Chen et al., 2022b), and contribute to metabolic functions (Higashi et al., 2023). Assembled through metabolic compatibility and comparable avirulent or pathogenic potential, oral taxa maintain dense metabolic, signaling, and tight spatial interactions, forming stable consortia. These consortia interact closely and tend to follow a defined temporal sequence of colonization during the development of oral diseases. Although their classification varies across the literature, these oral taxa are broadly grouped according to their ecological roles as commensals and beneficial symbionts, regarded as early colonizers of the oral cavity; opportunists; pathogens at higher loads, predominantly members of the mature biofilm (Socransky et al., 1998; Kolenbrander et al., 2010). Commensals such as *Streptococcus* spp (Zhang et al., 2008; Guo et al., 2014)., *Actinomyces* spp. (several taxa formerly classified within this genus have since been reassigned to other genera, including *Schaalia*) (Kaplan et al., 2009; Yachida et al., 2019), and *Gemella morbillorum* (Yachida et al., 2019) establish and support a healthy niche.

Opportunists are closely associated with stable biofilm formation (Kolenbrander et al., 2002; Mohanty et al., 2019), encompassing *Fusobacterium* sp (Wu et al., 2015; Chen et al., 2022b; Villar-Ortega et al., 2022)., *Selenomonas* sp (Debertin et al., 2023)., *Leptotrichia* sp (Warren et al., 2013)., *Prevotella* sp (Mohanty et al., 2019)., in particular *Prevotella intermedia* (Mohanty et al., 2019; Karched et al., 2022; Higashi et al., 2023), and *Parvimonas micra* (Chang et al., 2023; Higashi et al., 2023). Beyond these bacterial taxa, the opportunistic fungus *Candida albicans* warrants specific consideration as a critical cross-kingdom participant within these polymicrobial networks (Wu et al., 2015; Morse et al., 2019; Wronowska et al., 2025).

The established pathogenic consortium (Socransky et al., 1998; Mohanty et al., 2019) is dominated by *Porphyromonas gingivalis* (Guo et al., 2010; El-Awady et al., 2019; Mohanty et al., 2019), and *Treponema* sp (El-Awady et al., 2019; Mohanty et al., 2019; Oogai et al., 2025).

Since oral bacteria colonize the colon as coordinated communities, their native structural organization and temporal succession during colonization are directly relevant to CRC (Nakatsu et al., 2015; Drewes et al., 2017; Flemer et al., 2018; Yachida et al., 2019; Hu et al., 2025). After relocating from the oral cavity to the colon, even species that are harmless in their native oral niche may facilitate the establishment of oncogenic pathogens, and commensal and beneficial symbiont taxa can shift toward a pathobiont phenotype, driving tumor community progression (Flemer et al., 2018; Yachida et al., 2019; Löwenmark et al., 2024; Govindarajan et al., 2025). Some key microbial participants in carcinogenesis are detectable at CRC promotion and show consistent presence with progressive enrichment through advanced disease to metastatic sites (Bullman et al., 2017; Piccinno et al., 2025).

The presence of oral bacterial species within the colorectal cancer (CRC) tumor microenvironment is both widespread and spatially heterogeneous (Bullman et al., 2017). Invasive polymicrobial biofilms, actively colonized by oral pathogens, are present in approximately 50% of all CRC specimens and in 89–100% of right-sided colonic tumors (Dejea et al., 2014; Drewes et al., 2017; Avril and DePaolo, 2021). Notably, *Fusobacterium* species act as key structural taxa and are detectable in the majority of polymicrobial tumor biofilms (Drewes et al., 2017; Kvich et al., 2024; Zepeda-Rivera et al., 2024; Queen et al., 2025). Critically, most historical data regarding the species-level identification of Fusobacteria are based on short-read sequencing of 16S rRNA hypervariable regions, which lacks the resolution to distinguish individual species within the *Fusobacterium nucleatum* sensu lato (Löwenmark et al., 2024). The ongoing taxonomic debate, driven by the shift toward strain-level resolution in CRC studies, has revealed substantial genetic heterogeneity (Quagliariello et al., 2025). This shift has recently led to the reclassification of several former subspecies into distinct, independent species (Incognito et al., 2025; Sivertsen et al., 2025). For instance, earlier CRC studies may have reported *Fusobacterium animalis*-related strains under historical *F. nucleatum* nomenclature, including *F. nucleatum* EAVG_002/7/1 and *F. nucleatum* subsp. *animalis* strain 7/1 (Kostic et al., 2013; Cochrane et al., 2016; Zepeda-Rivera et al., 2024; Sivertsen et al., 2025). *Fusobacterium* spp. have been identified in 78.9% – 86.6% of all CRC lesions at varying relative abundances (Kvich et al., 2024; Queen et al., 2025). However, the contribution of the oral microbiome to carcinogenesis is not restricted to *Fusobacterium* species. Co-enrichment of tumors with other oral bacteria (e.g., *Pa. micra*, *Peptostreptococcus stomatis*) is observed in nearly 48% of cases (Drewes et al., 2017).

The involvement of oral bacteria in colorectal carcinogenesis can be interpreted through several complementary conceptual frameworks, specifically: through the analytical approach of co-abundance groups (CAGs) (Flemer et al., 2018; Yachida et al., 2019; Piccinno et al., 2025), through the structural-ecological concept of bridging organisms (Kolenbrander and London, 1993; Kaplan et al., 2009; Brennan and Garrett, 2019), and through the bacterial driver-passenger model (Tjalsma et al., 2012; Fidelle et al., 2020; Xing et al., 2022). While each framework captures a specific dimension of oral bacteria contribution in the pathological process, together they provide a comprehensive understanding of their role in tumor progression. The combination of these perspectives facilitates a crucial shift from a simplified focus on individual taxa and their isolated virulence determinants toward an understanding of the microbial community as a multidimensional ecosystem.

**Figure 1:**
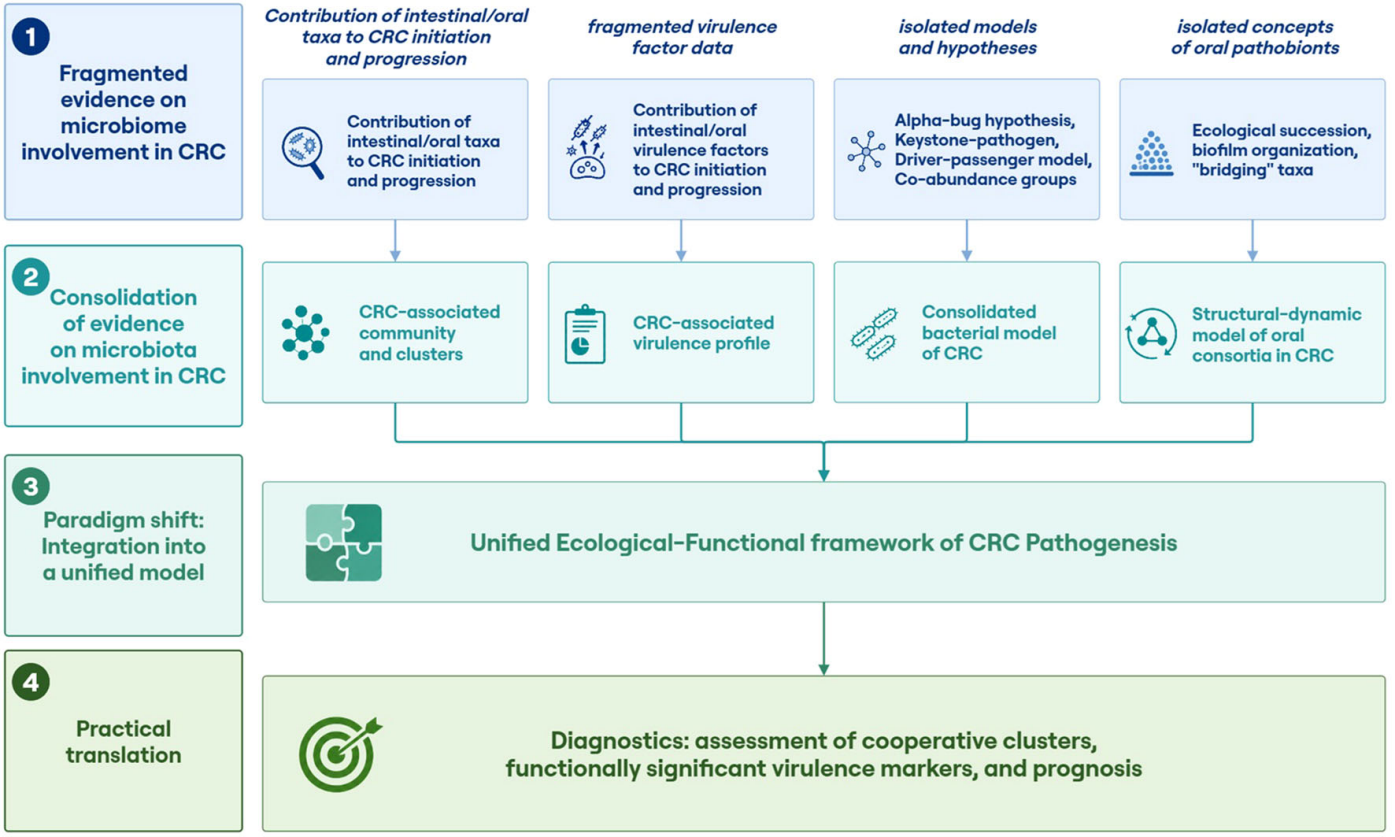
From fragmented oral microbiome evidence to an integrated ecological-virulome framework for CRC pathogenesis and risk stratification.

Previous reviews have separately addressed the oral-gut microbiome axis, individual CRC-associated oral taxa, microbial biofilms, various bacterial models and concepts, distinct intra-community processes associated with CRC, and selected virulence factors. The novelty of the present review does not lie in introducing these components individually, but in integrating them into a single community-level framework (see **Figure 1**).

Specifically, we relate the ecological succession and bridging organization of oral biofilms to the stage-dependent assembly of CRC-associated consortia and map the virulence determinants of individual taxa onto complementary functions distributed across the community. This approach shifts the unit of interpretation from the presence of an isolated taxon or virulence factor to a composite profile comprising taxonomic identity, strain-level virulence repertoire, ecological position, interaction partners, and disease context. We further discuss how this framework may guide the development and validation of multimarker risk-stratification models combining microbial taxa, cooperative clusters, and functionally relevant virulence determinants.

## Literature search strategy and selection criteria

This narrative review draws on a structured literature search aimed at identifying studies addressing the contribution of oral-associated microorganisms to CRC, with particular emphasis on polymicrobial interactions, bacterial translocation, bridging organisms, virulence determinants, and microbiome-based diagnostic and therapeutic implications. The search was conducted across PubMed/MEDLINE, Scopus, Web of Science, and Google Scholar, covering literature published up to June 2026. Priority was given to recent publications: the search period was restricted to the past 10 years for experimental studies and the past 5 years for classical reviews, systematic reviews, and meta-analyses. These temporal restrictions did not apply to seminal historical works addressing foundational concepts, key discoveries, or original findings of defining relevance to the scope of this review.

The search strategy combined terms related to CRC together with terms describing oral and gut microbiota, microbial translocation, biofilm ecology, virulence mechanisms, ecological and polymicrobial interactions, tumor-immune microenvironment and immune evasion, as well as microbial diagnostic biomarkers, chemoresistance, and responses to immunotherapy.

The literature search and selection process considered human studies, CRC animal models, *in vitro* and *ex vivo* mechanistic studies, and multi-omics analyses, all of which were eligible when relevant to the conceptual scope of the review. Meta-analyses were used as secondary evidence, primarily to support broader trends or diagnostic associations across studies. Review articles were used sparingly, mainly to provide background context or to identify additional primary studies, rather than as primary evidence.

Studies unrelated to CRC, oral-associated microorganisms, microbial translocation, polymicrobial interactions, or virulence mechanisms were excluded. Studies on non-CRC diseases were not eligible unless they provided mechanistic evidence directly relevant to oral bacterial translocation, bloodstream survival, coaggregation, biofilm formation, or virulence functions discussed in the CRC context.

## Microbial complexes in the oral cavity

In 1998, Sigmund S. Socransky and colleagues performed an empirical clustering of co-colonizing species across a large set of subgingival plaque samples. Based on cluster analysis and community ordination, microorganisms were grouped according to their co-occurrence patterns and correlated with the severity of periodontal manifestations into the yellow, green, purple, orange, and red complexes. The succession of these bacterial complexes within the oral cavity during biofilm formation has historically been interpreted within a model of ordered ecological replacement (Socransky et al., 1998; Kolenbrander et al., 2002; Kolenbrander et al., 2010). Rather than competitively displacing one another, bacteria interact synergistically: preceding groups establish attachment sites for subsequent colonizers. Early colonizers comprise yellow, green, and purple complexes, which possess the capacity for direct surface adhesion. These taxa establish the foundational biofilm layer and initiate alterations to the local microenvironment. The orange complex consists of intermediate colonizers capable of coaggregating with early-stage taxa, with *F. nucleatum* playing a pivotal role. The red complex includes aggressive late-stage colonizers. They are strict anaerobes, rarely detected in biofilms in the absence of orange complex bacteria. These taxa demonstrate particularly strong associations with increased periodontal pocket depth and gingival bleeding.

The yellow complex comprises species of the genus *Streptococcus*, among them *Streptococcus sanguis*, *Streptococcus oralis*, *Streptococcus mitis*, *Streptococcus gordonii*, and *Streptococcus intermedius.* The green complex incorporates *Campylobacter concisus, Eikenella corrodens*, and *Aggregatibacter actinomycetemcomitans* (formerly *Actinobacillus actinomycetemcomitans*). The purple complex includes representatives of the genera *Actinomyces*, species *Veillonella parvula*, and *Selenomonas noxia.* Species of the orange complex include members of the genera *Fusobacterium*, *Prevotella, Campylobacter*, as well as *Pa. micra, Eubacterium nodatum*, and *Streptococcus constellatus.* The species within the red complex are *Po. gingivalis*, *Treponema denticola* and *Tannerella forsythia* (Socransky et al., 1998; Mohanty et al., 2019).

Emerging evidence has challenged and expanded this framework, revealing that the actual diversity of clinically relevant taxa and their network interactions is considerably broader (El-Awady et al., 2019; Fernandes et al., 2024; Soueidan et al., 2024), and that the complexes do not function as monolithic and static units but rather exhibit dynamic compositional and functional flexibility (Morse et al., 2019; Sakanaka et al., 2022; Hall et al., 2023). Bacteria of the aggressive “red complex” co-occur and interact with taxa from all five complexes, including clinically healthy sites. Critically, polymicrobial synergy (mutual potentiation) (El-Awady et al., 2019), rather than quantitative shifts in abundance, drives the initiation of pathogenesis (Hajishengallis et al., 2012; Lamont and Hajishengallis, 2015; Fernandes et al., 2024). A key refinement to the original model is the observation that red complex bacteria may already be present during early inflammatory stages (but without a drastic population increase), and *Fusobacterium*-associated taxa may initiate the primary dysbiotic shift and trigger the inflammatory cascade, actively conditioning the microenvironment (Hall et al., 2023).

It can be confidently stated that the properties of oral bacteria, including their aggregation capacity, interspecies interaction patterns, interdependence within complexes, their defined roles, and the temporal dynamics of colonization during intestinal pathogenesis, exhibit remarkable parallels to their behavior within the oral niche (Drewes et al., 2017; Flemer et al., 2018; Piccinno et al., 2025).

## Oral taxa in gut co-abundance groups

Oral polymicrobial consortia (within contemporary expanded frameworks) exhibit striking analogies with CAGs in studies linking the gut microbiome to intestinal pathologies, including CRC (Dejea et al., 2014; Flemer et al., 2018; Horiuchi et al., 2020; Conde‐Pérez et al., 2024b; Conde‐Pérez et al., 2024a; Paduraru et al., 2025). CAGs represent dynamic bacterial clusters unified by functional and metabolic interdependence beyond mere physical proximity, characterized by synchronized abundance changes that form an integrated ecological network. Accumulating studies demonstrate that oral bacteria originating from dental plaque migrate to the intestine and act as intact communities within colorectal tumor specimens (Flynn et al., 2016; Flemer et al., 2018; Mohanty et al., 2019). This confirms that oral taxa do not colonize tumors randomly, but instead migrate and establish residence as coordinated, cohesive communities, mutually dependent for survival and synergistic amplification of pathogenic capacity (Warren et al., 2013; Nakatsu et al., 2015; Flynn et al., 2016; Drewes et al., 2017; Flemer et al., 2018; Sakanaka et al., 2022; Bautista et al., 2026). In the gut, they organize into tumor-resident CAGs that, in both composition and temporal dynamics, remarkably recapitulate the structure of oral biofilms (Mohanty et al., 2019; Galeano Niño et al., 2022; Liang et al., 2024), progressing from early colonizers to late-arriving pathogenic consortium members (Kolenbrander et al., 2010; Flemer et al., 2018).

## Refinement of the “bridging bacteria” concept

A fundamental feature of many oral taxa is their ability to form structurally stable heterotypic biofilms through interspecies interactions across diverse ecological contexts (Meuric et al., 2013; Barbosa et al., 2015; Lima et al., 2017; Dohlman et al., 2021). The formation, structural organization, and maturation of complex polymicrobial communities, as well as the physical linking of early and late colonizers that are otherwise unable to establish contact with one another or attach to surfaces, fundamentally depends on the participation of specific “bridging organisms” that function as central connectors (Kolenbrander and London, 1993; Kaplan et al., 2009; Wu et al., 2015; Lima et al., 2017; Brennan and Garrett, 2019).

The synergistic (Guo et al., 2014; Barbosa et al., 2015; Paduraru et al., 2025; Wronowska et al., 2025; Bautista et al., 2026) effects of physical, chemical, and metabolic interactions (Guo et al., 2014; Lima et al., 2017; Karched et al., 2022) within oral consortia are reflected in several functional outcomes, including:

- Resource exchange (Guo et al., 2014; Neilands et al., 2019; Horiuchi et al., 2020)
- Enhanced community survival (Guo et al., 2014; Kuboniwa et al., 2017; Horiuchi et al., 2020; Bautista et al., 2026)
- Virulence amplification (El-Awady et al., 2019; Morse et al., 2019; Karched et al., 2022; Wronowska et al., 2025)
- Translocation to ectopic niches (Bullman et al., 2017; El-Awady et al., 2019; Paduraru et al., 2025)
- Facilitated carriage of otherwise non-invasive species (Edwards et al., 2007; Zhang et al., 2008; Kaplan et al., 2009; El-Awady et al., 2019)
- Overall remodeling of the local microenvironment and promotion of pathological tissue alterations (Guo et al., 2014; Brennan and Garrett, 2019; Higashi et al., 2023).

Traditionally, the *F. nucleatum* sensu lato has been assigned the key bridging role, owing to its exceptional abundance of surface adhesins that enable it to interconnect diverse microbial taxa. This bacterium serves as a physical “bridge”, linking early gram-positive colonizers (such as *Streptococcus* and *Actinomyces* spp.) into a functional community with late gram-negative species (including *Po. gingivalis*, *T. denticola*, and *Prevotella* spp.), many of which lack the inherent capacity for autonomous surface attachment under natural conditions (Kaplan et al., 2009; Okuda et al., 2012; Wu et al., 2015; Oogai et al., 2025).

The impact of several representatives of *F. nucleatum* (and related oral *Fusobacterium* species (Guo et al., 2017)) on community stability is so substantial that this capacity represents a relevant therapeutic target. Targeted disruption of intra-biofilm interactions, such as blocking the bridging function of *F. nucleatum* sensu lato (Wang and Fang, 2023; Kvich et al., 2024; Paduraru et al., 2025; Jung, 2026) or competitive inhibition of coaggregation (validated in mechanistic studies on specific strains like *F. nucleatum sensu stricto* (Wu et al., 2015) and *Fusobacterium polymorphum* (Edwards et al., 2006) has the potential to destabilize and dismantle the entire carcinogenic consortium. Multiple experimental studies report cross-colonization synergy and interspecies coaggregation, signaling, and horizontal gene transfer between *F. nucleatum* and other oral taxa, including related *Fusobacterium* species (Crowley et al., 2024), *Leptotrichia* sp (Warren et al., 2013; Dohlman et al., 2021)., *Selenomonas* sp (Warren et al., 2013)., *Pa. micra* (Horiuchi et al., 2020; Löwenmark et al., 2024)*, Po. gingivalis* (Kinder and Holt, 1993; Okuda et al., 2012; Brennan and Garrett, 2019; Govindarajan et al., 2025; Li et al., 2025)*, Peptostreptococcus stomatis* (Drewes et al., 2017)*, Pr. intermedia* (Okuda et al., 2012)*, T. denticola* (Meuric et al., 2013; Govindarajan et al., 2025)*, Actinomyces naeslundii* (Kaplan et al., 2009)*, Actinomyces oris* (Bibek and Wu, 2025), *Ag. actinomycetemcomitans* (Warren et al., 2013), *Cn. albicans* (Wu et al., 2015; Bibek and Wu, 2025), *S. oralis* (Kaplan et al., 2009; Bibek and Wu, 2025)*, S. sanguinis* (Kaplan et al., 2009; Zhang and Rudney, 2011; Kaplan et al., 2014; Bibek and Wu, 2025), *S. gordonii* (Kaplan et al., 2009; Kaplan et al., 2014; Lima et al., 2017; Bibek and Wu, 2025)*, Streptococcus cristatus* (Edwards et al., 2006; Bibek and Wu, 2025), *Streptococcus mutans* (Bibek and Wu, 2025), and *Campylobacter showae* (Warren et al., 2013).

Similarly, *Pa. micra* represents another microorganism with comparable bridging potential, possessing robust interspecies coaggregation mechanisms (Neilands et al., 2019; Horiuchi et al., 2020; Higashi et al., 2023; Löwenmark et al., 2024) and the capacity for autonomous attachment to epithelial surfaces, including intestinal mucosa (Chang et al., 2023). Likewise, *Po. gingivalis* displays multiple functional attributes of a bridging organism through active participation in cross-taxa physical and chemical interactions (Amano et al., 1997; Maeda et al., 2004; Guo et al., 2010; Meuric et al., 2013; Takasaki et al., 2013; Guo et al., 2014; Alaei et al., 2019; El-Awady et al., 2019).

*Cn. albicans* does not possess all characteristics of a classical “bridge”. It can be conceptualized as an interspecies structural scaffold that organizes surrounding bacterial communities and actively interacts with bridging organisms, early colonizers, and late-stage taxa. *Cn. albicans* provides extensive surface (hyphal networks), organizes bacterial attachment, and creates protective, anaerobic microenvironments conducive to survival and cooperative pathogenesis (Cruz et al., 2013; Schlecht et al., 2015; Wu et al., 2015; Ren et al., 2022; Bibek and Wu, 2025; Wronowska et al., 2025). Recently, it was shown that *Cn. albicans* physically transports *F. nucleatum* into colorectal tumors via Flo9-RadD interaction, synergistically accelerating CRC progression through amplified NF-κB signaling and immunosuppression (Li et al., 2026).

Importantly, a “bridging organism” is not necessarily a discrete taxonomic entity, but rather a functional role within the oral community. This function of consortium coordination proves critical during systemic dissemination of oral pathogens (Dohlman et al., 2021; Kudra et al., 2023; Löwenmark et al., 2024). The structural-ecological concept of bridging organisms is essential for interpreting the interconnections between the oral cavity and diverse ecological niches during organism translocation. Individual species may function as “transitional” or “connective” elements within consortia, and the evolutionary advantages enabling survival in the oral niche can drive pathological processes in distant sites (Amano et al., 1997; Maeda et al., 2004; Okuda et al., 2012; Meuric et al., 2013; Takasaki et al., 2013; Barbosa et al., 2015; Alaei et al., 2019; Neilands et al., 2019; Horiuchi et al., 2020; Löwenmark et al., 2024).

## Microbial complexes and bridging taxa within the driver-passenger model

### Oral-gut succession in the driver-passenger model

The classical driver-passenger model (Tjalsma et al., 2012) posits that certain organisms initiate tissue damage and primary carcinogenic alterations, with others subsequently colonizing the modified niche at later stages. The driver-passenger model conceptually mirrors the framework of oral complexes and early-to-late colonizer succession in the oral niche. In both cases, the mature pathogenic consortium formation rests on a common principle: certain microorganisms prepare the structural and metabolic landscape, allowing others to colonize it and maintain chronic disease. The adaptive characteristics of CRC-associated oral taxa are uniquely equipped to exploit the pathological and dysbiotic conditions of the malignant intestinal microenvironment rather than a healthy colon (Flynn et al., 2016; Mathlouthi et al., 2022; Chang et al., 2023; Löwenmark et al., 2024). Indigenous intestinal primary drivers are frequently displaced by oral pathobionts, including *Fusobacterium* species, *Pa. micra*, *Po. gingivalis*, and *Prevotella* spp., which, as evidenced by co-abundance networks (Flemer et al., 2018) and *in vitro* models, appear to behave similarly to their role in oral disease (Flynn et al., 2016; Chang et al., 2023; Löwenmark et al., 2024).

Traditionally, canonical representatives of the oral microbiota, such as *F. nucleatum* sensu lato, *Pa. micra*, *Ps. stomatis*, and *Po. gingivalis* were classified merely as classic “passengers” (Tjalsma et al., 2012; Garza et al., 2020; Wang et al., 2020; Tremblay et al., 2021; Higashi et al., 2023). Within the *Fusobacterium* species, the CRC-relevant contributions of *F. nucleatum* and *F. animalis* (previously *F. nucleatum* subsp. *animalis* clade C2 (Sivertsen et al., 2025)) are best characterized. Their abundance and prevalence increase markedly at advanced disease stages (III and IV) and during metastasis (Mathlouthi et al., 2022). However, recent data from *in vitro* experiments (including cell lines, co-cultures, and human organoids) (Kostic et al., 2013; Guo et al., 2020; Mu et al., 2020; Bergsten et al., 2023; Motosugi et al., 2025; Nguyen et al., 2025), *in vivo* animal models (Kostic et al., 2013; Guo et al., 2020; Motosugi et al., 2025; Nguyen et al., 2025), and associative mechanistic studies on human material (Kostic et al., 2013; Bullman et al., 2017; Wang et al., 2021; Bergsten et al., 2023; Wang and Fang, 2023; Motosugi et al., 2025) suggest that these oral pathobionts demonstrate tumor-promoting properties and may contribute to tumor progression and acceleration (for further details, see **Supplementary Table 1**). Empirical observations demonstrate highly complex CRC-associated polymicrobial interactions involving functionally intermediate participants (Löwenmark et al., 2024; Glazunova et al., 2025). These taxa establish conditions under which the carcinogenic consortium becomes increasingly active (Engevik et al., 2021), stable (Dohlman et al., 2021; Engevik et al., 2021; Xing et al., 2022; Bautista et al., 2026), competitive (Drewes et al., 2017; Dohlman et al., 2021; Löwenmark et al., 2024; Hu et al., 2025), and resistant (Dohlman et al., 2021; Engevik et al., 2021; Xing et al., 2022; Bautista et al., 2026). An essential aspect of this new paradigm shift is the conceptualization of polymicrobial biofilms, enriched with these oral species, as potential functional drivers of oncogenic transformation (Tomkovich et al., 2019; Cheng et al., 2020; Glazunova et al., 2025; Kushwaha et al., 2025).

### Bridging taxa as structural anchors of the CRC-associated microbial complex

The formal application of the “bridging organism” concept to the CRC niche requires explicit definitional criteria to distinguish functional potential from validated ecological roles *in vivo*. Specifically, a qualifying taxon must: (i) demonstrate physical bridging capacity and the formation or maintenance of a biofilm scaffold within the CRC niche; (ii) facilitate the colonization of taxa lacking autonomous surface attachment; and (iii) show confirmed spatial co-localization within human CRC tissue alongside evidence of synergistically enhancing or maintaining consortium virulence. Additionally, the metabolic facilitation of microbial succession may be considered an additional, though debatable, feature of bridging taxa.

In the driver-passenger model context, the role of proposed oral bridging species requires broader interpretation. Beyond the established primary bridging functions, *F. nucleatum* serves as a critical biological scaffold for colorectal carcinogenesis, actively promoting and sustaining malignant tumor growth, as demonstrated by preclinical models (Kostic et al., 2013; Mima et al., 2016; Bullman et al., 2017; Rubinstein et al., 2019). Meanwhile, other potential bridging taxa assist in maintaining microbial community succession both during the promotion and progression stages of carcinogenesis (Kinder and Holt, 1993; Edwards et al., 2006; Kaplan et al., 2009; Zhang and Rudney, 2011; Okuda et al., 2012; Warren et al., 2013; Kaplan et al., 2014; Lima et al., 2017; Brennan and Garrett, 2019; Dohlman et al., 2021; Bibek and Wu, 2025; Govindarajan et al., 2025; Li et al., 2025). Within the CRC literature, the terms “bridging organism” and “bridging species” are used primarily as terminology adapted from ecology to illustrate how certain taxa bridge early and late colonizers to stabilize polymicrobial biofilms (Drewes et al., 2017; Choudhury et al., 2025; Li et al., 2025; Paduraru et al., 2025). The concept has not been established in oncology as an independent classificatory category to describe a group of structural stabilizers (Paduraru et al., 2025).

Applying explicit previously defined criteria for bridging organisms enables evidence-based stratification of proposed bridging taxa. *F. nucleatum* sensu lato and *F. animalis* strains currently meet these requirements, supported by robust experimental evidence from human CRC tissue (Dejea et al., 2014; Abed et al., 2016; Bullman et al., 2017; Drewes et al., 2017; Rubinstein et al., 2019; Kvich et al., 2024). In contrast, direct evidence that *Pa. micra* and *Po. gingivalis* physically link early and late colonizers within the CRC tumor microenvironment remains limited, derived primarily from oral biofilm *in vivo* or *in vitro* models (Amano et al., 1997; Maeda et al., 2004; Alaei et al., 2019; Neilands et al., 2019; Horiuchi et al., 2020). *Pa. micra*, *Po. gingivalis*, and *Prevotella* spp. demonstrate substantial functional potential for synergistic biofilm formation and interspecies coaggregation, but their direct bridging roles within the human CRC niche await direct spatial validation. For instance, *Pr. intermedia* has been shown to be enriched in colorectal adenocarcinomas, co-abundant with *F. nucleatum* in CRC tissue, and to exert a synergistic additive effect on cancer cell invasion (Lo et al., 2022). These findings support the functional potential of *Pr. intermedia* but do not validate its ecological role *in vivo*. Previously considered speculative, the role of *Cn. albicans* as a cross-kingdom scaffold has now been definitively established *in vivo* (Li et al., 2026).

Nevertheless, introducing the concept of bridge species to the CRC field offers a promising framework to elucidate their critical role in pathogenesis and to position them as key diagnostic markers (Drewes et al., 2017; Flemer et al., 2018; Löwenmark et al., 2024).

### Mechanisms of oral pathogen translocation to the intestine

Oral bacteria selectively localize and colonize colorectal tumors as stable, intact consortia rather than as individual cells, through specific tropism for malignant intestinal epithelial cells (Warren et al., 2013; Bullman et al., 2017; Drewes et al., 2017). These complex associations persist even in distant metastatic lesions (Bullman et al., 2017).

Two primary routes mediate oral bacterial translocation to the intestine. The distinct physiological and biochemical barriers inherent to each route act as selective “filters”, determining which taxa and even which strains survive passage.

The first pathway is the direct (via the gastrointestinal tract) enteral pathway (Kostic et al., 2013; Komiya et al., 2019), where only microorganisms capable of adapting to extreme conditions survive: gastric acidity (Zepeda-Rivera et al., 2024) and the toxic effects of bile salts and digestive enzymes (Strain et al., 2022; Rashidi et al., 2023). *F. animalis* possess the capacity to overcome gastric acidity (pH 3) and establish intestinal colonization (Zepeda-Rivera et al., 2024).

Similarly, *in vivo* animal models demonstrate that oral *Ps. anaerobius* and *Po. gingivalis* successfully establish colonic colonization via this direct route and promote tumorigenesis (Long et al., 2019; Wang et al., 2021; Motosugi et al., 2025). Furthermore, *Schaalia odontolytica* (formerly *Actinomyces odontolyticus*) (Nouioui et al., 2018) and certain *Streptococcus* spp. can overcome the gastrointestinal tract barriers (see **Supplementary Table 1**) (Yachida et al., 2019; Lamaudière et al., 2023; Rashidi et al., 2023). Enteral transmission presents not only chemical stress but also intense competitive pressure for stable intestinal colonization (Queen et al., 2022). While human tissue tracing provides definitive *in vivo* evidence of clonal translocation of *Pa. micra* from the oral cavity to the colorectal tumor (Conde‐Pérez et al., 2024b), the precise route of this dissemination (whether via direct enteral transit or hematogenous spread) remains unresolved.

The second route is hematogenous dissemination via transient bacteremia resulting from mucosal barrier disruption and impaired perfusion. The systemic influx of oral pathogens into the bloodstream is critically amplified by inflammatory diseases like periodontitis and odontogenic abscesses, where ulcerated tissues act as direct portals for vascular entry (Abed et al., 2016; Brennan and Garrett, 2019; Abed et al., 2020). *Po. gingivalis* and several species of *Streptococcus* may survive bloodstream transit by invading peripheral blood mononuclear cells, which potentially serve as delivery systems to distant sites, including colorectal tumors (El-Awady et al., 2019). Additionally, *Pa. micra* demonstrated marked preference for the hematogenous route (Watanabe et al., 2020; Higashi et al., 2023). In murine models, *F. animalis* and *F. nucleatum* preferentially utilized the hematogenous route (Abed et al., 2020; Queen et al., 2022). Nevertheless, current literature also provides substantial evidence for the enteral pathway as a direct spread of oral *F. nucleatum* sensu lato translocation to colorectal tumor tissue (Kostic et al., 2013; Wang and Fang, 2023).

The “vector-mediated” mechanism warrants attention. For taxa lacking either resistance mechanisms for enteral transit or capacity for systemic survival in blood, an alternative route exists through coaggregation. The biological feasibility of this mechanism is supported by clinical evidence based on whole-genome and metatranscriptomic sequencing of patient samples, which revealed consistent co-occurrence and co-aggregation networks (Warren et al., 2013; Flynn et al., 2016). Furthermore, this concept is functionally validated by evidence derived from *in vitro* co-aggregation experiments within CRC tumors (Warren et al., 2013). This process is exemplified by the transport of *S. cristatus, S. sanguinis* and *Ac. naeslundii* by *F. polymorphum* (Edwards et al., 2006).

*Fusobacterium* species, as primary bridging organisms of the oral consortium, can transport and establish other microorganisms (*Porphyromonas* spp., *Peptostreptococcus* spp., *Parvimonas* spp.) lacking independent dissemination capacity, functioning as “vectorial” carriers, as shown *in vitro* (Warren et al., 2013; Flynn et al., 2016). *F. nucleatum* sensu lato invades epithelial cells and effectively translocates attached non-invasive taxa into host cell cytoplasm as demonstrated in *in vitro* experiments (Edwards et al., 2006). This mechanism is also observed at the interkingdom level, with *Cn. albicans* functioning as the major vector organism capable of translocating attached bacteria across mucosal barriers (Schlecht et al., 2015; Sprague et al., 2024) or facilitating their spatial spread across surfaces (*in vitro*) (Ren et al., 2022).

### Virulence determinants of oral taxa in the context of CRC

Although oral species were identified as tumor-enriched colonizers, the absence of canonical exotoxins led to their long-standing classification as merely opportunistic passengers. However, accumulating experimental data on the oral virulome and the mechanisms by which individual virulence determinants interact with intestinal cells have repositioned several of these taxa as active contributors to carcinogenesis. This repositioning should be interpreted at strain- and lineage-level resolution, as virulence determinants are not uniformly present or functionally equivalent across all members of a given taxon. Their distribution may be species-, subspecies-, lineage-, or strain-specific, with activity varying by gene sequence, copy number, regulation, expression, and ecological context. Taxon-level enrichment in CRC samples therefore does not necessarily imply presence or activity of the full virulence repertoire. The following section accordingly discusses virulence factors as determinants of specific taxa, lineages, or strains where evidence permits, rather than as universal properties of entire genera or species. Key virulence factors of oral taxa are pleiotropic and can be categorized into five functional groups.

The first group comprises determinants of interbacterial interaction and cooperation, facilitation of bacterial colonization, and surface adhesion, contributing to biofilm formation and modulation. This group includes coaggregation and biofilm regulation factors such as: FadA, FadA2, FadA3, RadD, Fap2, Aid1, and CmpA (*F. nucleatum* sensu lato) (Kaplan et al., 2009; Kaplan et al., 2014; Guo et al., 2017; Lima et al., 2017; Bibek and Wu, 2025), Als3 (*Cn. albicans*) (Silverman et al., 2010; Schlecht et al., 2015; Sztukowska et al., 2018; Wronowska et al., 2025), FimA, RgpA, RgpB, and Kgp (*Po. gingivalis*) (Guo et al., 2014; Govindarajan et al., 2025; Wronowska et al., 2025), PCWBR2 experimentally characterized in *Ps. anaerobius* (Long et al., 2019), and putative PCWBR2 annotated in *Ps. stomatis* DSM 1 (Putative cell wall binding repeat 2 [Peptostreptococcus stomatis DSM 17678). *F. nucleatum sensu stricto* coaggregates via its adhesins not only with a broad range of oral species but also with resident intestinal taxa: *Enterococcus faecalis* (Kaplan et al., 2014) and *Clostridioides difficile* (Engevik et al., 2021). The *Fusobacterium* adhesin Fap2 selectively binds Gal-GalNAc receptors, which are overexpressed on cancer cells (Abed et al., 2016).

The second functional category is directed towards invasion and barrier disruption. Virulence factors such as FadA, FadA2, and FadA3 (Van Der Post et al., 2013; Govindarajan et al., 2025), potentially PCWBR2 of *Ps. stomatis* (Putative cell wall binding repeat 2 [Peptostreptococcus stomatis DSM 17678; Long et al., 2019) and gingipains (RgpA, RgpB, and Kgp) are involved in the degradation of the protective mucus layer and epithelial barrier (Van Der Post et al., 2013; Guo et al., 2020; Govindarajan et al., 2025) and the disruption of intestinal epithelial integrity (Drewes et al., 2017; Chen et al., 2022b; Wronowska et al., 2025; Bautista et al., 2026).

The third group focuses on niche formation through immune evasion, immunosuppression, and inflammatory modulation. Virulence factors such as Aim1, Aid1, RadD, CmpA, and Fap2 of *Fusobacterium* species, along with gingipains of *Po. gingivalis*, and potentially PCWBR2 of *Ps. stomatis* (Putative cell wall binding repeat 2 [Peptostreptococcus stomatis DSM 17678; Long et al., 2019) exert a broad spectrum of pathological effects on the host. These determinants induce inflammatory responses and promote the proteolytic degradation of antimicrobial peptides (Guo et al., 2010; Wronowska et al., 2025), and immune components, including immunoglobulins, complement factors, ICAM-1, and various cytokines (Govindarajan et al., 2025; Wronowska et al., 2025). Finally, these factors protect the pathogenic consortium by mediating profound immunosuppression through the direct inhibition and apoptosis of T-cells and natural killer (NK) cells (Kaplan et al., 2009; Guo et al., 2010; Tjalsma et al., 2012; Dejea et al., 2014; Wang et al., 2020; Engevik et al., 2021; Sakanaka et al., 2022; Paduraru et al., 2025).

The fourth functional group involves indirect genotoxicity, mediated by FadA, FadA2, and FadA3 of selected *Fusobacterium* species (Guo et al., 2020).

The fifth functional category encompasses factors responsible for the metabolic and structural changes of the tumor microenvironment (TME): induction of local hypoxia, extracellular matrix remodeling, promotion of tumor invasion, physical niche compartmentalization, intracellular signaling reprogramming, stimulation of proliferation, facilitation of metastatic dissemination, and apoptosis evasion. This capacity is attributed to RadD, Fap2, FadA, FadA2, and FadA3 (Kostic et al., 2013; Drewes et al., 2017; Rubinstein et al., 2019; Li et al., 2021; Meng et al., 2021; Incognito et al., 2025; Li et al., 2025; Bautista et al., 2026), potentially PCWBR2 (Long et al., 2019; Huang et al., 2024), and Als3 (Zhou et al., 2025).

Virulence potential data for several classic co-colonizer taxa remain limited, and their roles may require reassessment. For instance, *Pr. intermedia* harbors ~30 predicted virulence proteins potentially relevant to CRC association (Karched et al., 2022). *Pa. micra* displays gingipain-like activity accelerating CRC development, though the responsible virulence determinant remains unidentified (Neilands et al., 2019; Zhao et al., 2022).

Notably, classical oral pathobionts primarily employ cooperative strategies for successful colonization, lacking factors specifically directed toward the destruction of the indigenous gut microbiota (Neilands et al., 2019; Horiuchi et al., 2020; Sakanaka et al., 2022; Higashi et al., 2023; Li et al., 2025). In contrast, CRC-associated intestinal species possess distinct virulence factors designed to actively suppress microbial competitors (Kohoutova et al., 2014; He et al., 2019; Kiruthiga et al., 2020; Harrington et al., 2021; Li et al., 2022; Proutière et al., 2023; Tozzi et al., 2025).

To provide a comprehensive overview, we summarized data on 14 key virulence factors from the major oral taxa in **Supplementary Table 2**.

## Oral and gut-resident microbiota coexistence and ecological interactions

The intestinal CRC-associated microbiota include several species of *Streptococcus*, *Bacteroides*, *Campylobacter*, *Peptostreptococcus*, *Enterococcus*, *Salmonella*, *Shigella*, *Citrobacter*, *Cronobacter*, *Klebsiella*, *Enterobacter*, *Morganella*, as well as *Ag. actinomycetemcomitans* and *Cl. difficile*.

In the context of CRC development and progression, oral bacteria frequently link early and late participants within the gut microbiota, facilitating their epithelial adhesion and contributing to immune evasion, promoting inflammatory progression, disruption of intercellular junctions, and the breakdown of epithelial barrier integrity (Löwenmark et al., 2024; Sprague et al., 2024). For instance, *Pa. micra*, *F. nucleatum* sensu lato, and enterotoxigenic *Bacteroides fragilis* (ETBF) form a unified mucosal biofilm, particularly in tumors with microsatellite instability (MSI), where these species act as central hubs (Löwenmark et al., 2024). This consortium incorporates several other CRC-associated taxa: members of the genus *Eubacterium*, *Ps. stomatis, Parabacteroides distasonis*, and several uncharacterized species of the genus *Porphyromonas.*

*F. nucleatum* engages in complex interactions with the intestinal pathogen *Cl. difficile*. It chemically attracts *Cl. difficile*, and its surface adhesin RadD directly binds *Cl. difficile* flagella, activates virulence and defense mechanisms in *Cl. difficile.* The resulting biofilm architecture becomes significantly more resistant to host defense enzymes (such as DNase and proteinase K) as well as to antibiotic therapy, including vancomycin (Engevik et al., 2021; Chen et al., 2022b).

Similarly, *F. nucleatum sensu stricto* co-aggregates with the CRC-associated pathogen *E. faecalis* via the surface protein Aid1 (Kaplan et al., 2014). It is also capable of establishing mutually beneficial synergistic interactions with gut-resident ETBF and *pks*+ *Escherichia coli* even without obligate physical contact (Bautista et al., 2026). Acting in concert, these pathogens coordinately degrade the intestinal mucosa, induce host DNA mutations, and establish a persistent pro-oncogenic inflammatory milieu (Bautista et al., 2026).

**Figure 2:**
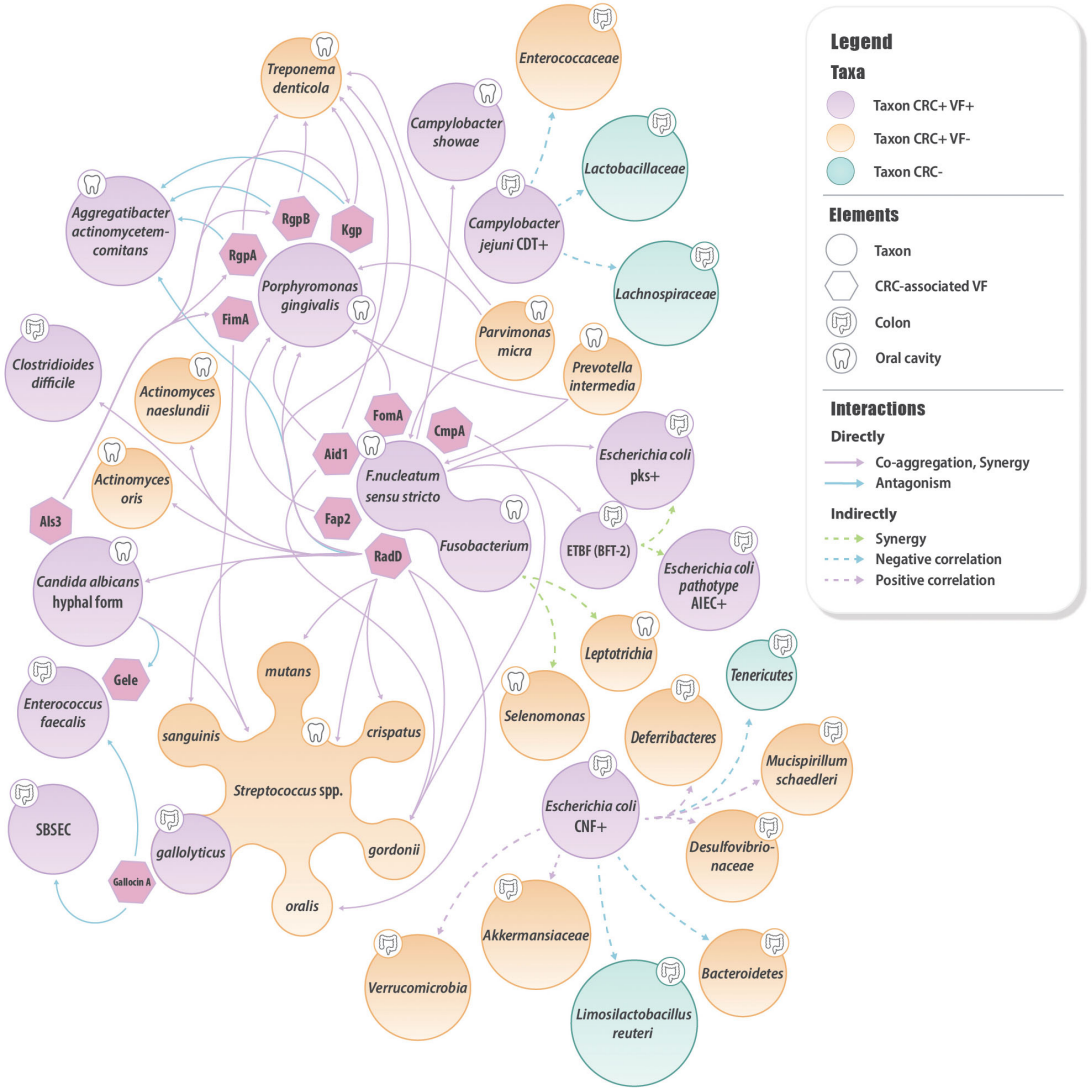
Interaction network of CRC-associated oral and intestinal taxa and their virulence factors. Circular nodes represent CRC-associated microbial taxa; hexagonal nodes represent CRC-associated virulence factors (VF). Taxa are color-coded according to their CRC association and virulence factor status: violet nodes indicate CRC-enriched taxa harboring characterized virulence factors (CRC+, VF+); orange nodes represent CRC-enriched taxa without currently characterized virulence factors (CRC+, VF-); blue-green nodes denote taxa with negative CRC association (CRC-). Tooth icons indicate taxa of predominantly oral origin; intestinal icons indicate gut-resident taxa, though some may span both niches. Direct interactions are shown as solid lines: purple arrows denote coaggregation and synergistic interactions; blue arrows indicate antagonistic relationships. Indirect associations are represented by dashed lines: green indicates indirect synergy; pink dashed lines represent negative correlations; blue dashed lines indicate positive correlations between taxa.

Identified synergistic and antagonistic interactions and correlations among CRC-associated species are illustrated in **Figure 2**.

## Limitations and future directions

### The challenge of causal directionality

Despite accumulating evidence linking oral pathobionts to colorectal carcinogenesis, causal directionality remains a fundamental unresolved question in microbiome research. The observed microbial enrichment in CRC may be explained by three alternative interpretations. First, specific oral taxa may directly promote CRC progression and drive pro-oncogenic inflammation. Second, enrichment of oral taxa may represent a consequence of niche remodeling during tumor progression. The local tumor microenvironment (characterized by hypoxia, necrosis, and metabolic shifts) creates conditions permissive for secondary colonization by opportunistic oral pathogens. Finally, certain microbial alterations may represent a coincidental bystander effect, reflecting systemic changes in host diet, immune status, or intestinal transit rather than active contributions to tumor progression. Distinguishing between these scenarios is critical. While preclinical models support active tumor-promoting roles for *F. nucleatum* and *Pa. micra*, establishing primary causation in humans requires longitudinal clinical evidence.

### Methodological limitations in translocation and resolution

While *in vivo* evidence of clonal translocation in humans has been established for key taxa such as *F. nucleatum* and *Pa. micra*, data for the broader consortium still rely largely on animal and *in vitro* models, which cannot fully recapitulate the human CRC microenvironment. Furthermore, even when translocation is confirmed *in vivo*, existing methodologies often fail to definitively differentiate between direct enteral transit and hematogenous dissemination routes.

### Gaps in spatial and functional validation

The functional and spatial roles of most proposed consortium members remain incompletely validated. For most candidate bridging or scaffold organisms, available data support functional compatibility rather than confirmed roles in human tumor tissue. Co-abundance and correlation networks do not equate to demonstrated physical interaction *in vivo*.

### Clinical implications

The presence of specific oral pathogens directly correlates with poor prognosis and treatment resistance. A distinct correlation is observed between specific consensus molecular subtypes (CMS) and their clinicopathological features, including prognosis, chemosensitivity, mutational status, and preferred anatomical location (Guinney et al., 2015; Fidelle et al., 2020; Karaman et al., 2025; Paduraru et al., 2025). Long-term observations similarly demonstrate a positive correlation between these clinical manifestations and the presence of certain subtype-associated taxa (Higashi et al., 2023; Shi et al., 2025).

For instance, the enrichment of *Fusobacterium* genus (and *F. nucleatum* in particular), exhibits a strong link with poor disease prognosis (Flanagan et al., 2014; Mima et al., 2016; Bullman et al., 2017; Yu et al., 2017; Rubinstein et al., 2019; Fidelle et al., 2020; Dougherty and Jobin, 2023; Löwenmark et al., 2024; Hu et al., 2025), metastasis (Warren et al., 2013; Abed et al., 2016; Bullman et al., 2017; Li et al., 2021), right-sided tumor localization (Flanagan et al., 2014; Mima et al., 2016; Bullman et al., 2017; Drewes et al., 2017; Raskov et al., 2018; Zwinsová et al., 2021; Kvich et al., 2024), chemoresistance (Onozawa et al., 2017; Yu et al., 2017; Rubinstein et al., 2019; Zhang et al., 2019), resistance to immunotherapy (Abed et al., 2016), MSI (Ito et al., 2015; Mima et al., 2016; Lee et al., 2021; Löwenmark et al., 2024), and a CpG island methylator phenotype-positive profile (Ito et al., 2015). Similarly, the presence of *Pa. micra* and *Ps. stomatis* (Löwenmark et al., 2024) correlates with the mutational status of tumor cells, specifically concerning the *KRAS* (Flanagan et al., 2014) and *BRAF* genes (Ito et al., 2015; Mima et al., 2016).

### Oral pathobionts as clinical markers

Oral bacteria, primarily *F. nucleatum* sensu lato (Viljoen et al., 2015; Drewes et al., 2017; Eklöf et al., 2017; Liang et al., 2017; Wong et al., 2017; Flemer et al., 2018; Guo et al., 2018; Osman et al., 2021), *Parvimonas* sp. including *Pa. micra* (Drewes et al., 2017; Flemer et al., 2018; Löwenmark et al., 2020; Osman et al., 2021; Shen et al., 2021), *Ps. stomatis* (Drewes et al., 2017; Thomas et al., 2019; Yachida et al., 2019; Wang et al., 2020; Osman et al., 2021; Shen et al., 2021), *Porphyromonas* (Thomas et al., 2019; Yachida et al., 2019; Wang et al., 2020; Löwenmark et al., 2024), and *Streptococcus* spp (Flemer et al., 2018). have emerged as independent or complementary biomarkers for CRC across multiple biological specimens via both invasive and non-invasive approaches. These taxa can be effectively detected through the screening of intestinal mucosal biopsies (Viljoen et al., 2015; Drewes et al., 2017; Wang et al., 2020), feces (Eklöf et al., 2017; Liang et al., 2017; Wong et al., 2017; Guo et al., 2018; Löwenmark et al., 2020; Wang et al., 2020; Osman et al., 2021; Shen et al., 2021; Piccinno et al., 2025), and even the oral cavity, where microbial signatures allow for CRC detection (Flemer et al., 2018; Komiya et al., 2019; Zhang et al., 2020), as well as combined multi-site analysis (Flemer et al., 2018; Cheng et al., 2020).

However, the clinical translation of oral microbiome-based CRC markers is limited by the low specificity of individual taxa and virulence determinants, many of which are enriched across multiple inflammatory and neoplastic conditions rather than CRC specifically. Therefore, relying solely on individual pathobionts, such as members of the *F. nucleatum* sensu lato or *Pa. micra*, may carry a high risk of false-positive results, particularly in patients with periodontal disease, inflammatory bowel disease (IBD), adenomas, or other gastrointestinal malignancies. Their performance should therefore be tested in well-defined cohorts that include not only healthy controls but also patients with the abovementioned diseases.

### Virulence factors and community succession patterns as clinical markers in diagnostics

The growing body of evidence on the role of oral taxa and their virulence factors in CRC calls for a paradigm shift in diagnostics. Traditional detection of individual bacterial taxa in isolation from their functional properties, community context, and succession dynamics is insufficient. Such an approach may fail to reflect the true oncogenic potential of the microbiota at a specific stage of disease progression, as bacteria do not act in isolation but rather within complex ecological networks. Their pathogenicity may depend on cooperative interactions at both the taxonomic and virulence-determinant levels, as well as on spatial organization and adaptive community remodeling.

Microbiome-associated carcinogenesis can be interpreted within the extended driver-passenger model, which describes the stepwise assembly of a carcinogenic community and provides a conceptual basis for explaining the role of oral species succession. Within this framework, bridge species and their virulence factors may serve as candidate markers of carcinogenesis and risk across various stages of CRC. Because the composition of the translocated oral consortium changes gradually within the colorectal niche, successive consortium members and their associated virulence determinants may serve as indirect indicators of colorectal cancer progression. However, these signatures require prospective validation before they can be interpreted as causal or clinically actionable markers.

Future diagnostic models should therefore move beyond single-marker detection and be validated as multi-parameter panels that integrate taxonomic profiles, cooperative microbial clusters, virulence-factor repertoires, sample type, and clinical context. A key priority is strain-level and virulome-resolved profiling, since species- or genus-level classification may obscure clinically relevant heterogeneity and CRC-associated properties can vary substantially across subspecies, lineages, and individual strains. Future studies should therefore target functional modules underlying adhesion, invasion, immune modulation, epithelial barrier disruption, and biofilm organization rather than taxonomy alone. Non-invasive screening should rely on the simultaneous detection of specific pathobionts, their clusters, and associated virulence factors. When selecting marker taxa, the diagnostic focus should shift toward identifying key bridge species, structural stabilizers that facilitate the formation of invasive polymicrobial biofilms, and species exhibiting similar bridging potential.

To improve CRC diagnostic specificity and distinguish a true CRC-associated community from incidental dysbiosis, preliminary multi-site profiling may be useful during biomarker development and validation. Combined oral and fecal profiling is particularly relevant for non-invasive risk stratification, whereas tissue-inclusive approaches are more appropriate for confirming tumor localization, spatial organization, epithelial invasion, crypt colonization, and preservation of polymicrobial community structure in the tumor niche. Correlating microbial and virulome signatures across saliva, stool, and mucosal or tumor tissue may support confirmation of pathogenic translocation and active tumor colonization during validation studies.

Importantly, future work should shift from associative description toward causally oriented, spatially resolved, and functionally validated models of oral – gut translocation. This requires longitudinal cohorts enrolled before CRC onset, with serial sampling across the adenoma – carcinoma pathway and parallel profiling of the mucosal or tumor metabolome and immune microenvironment. Such designs would help distinguish transient oral microbial carriage, disease-associated dysbiosis, selective enrichment by the tumor niche, and microbial patterns that precede neoplastic progression. Once an optimal biomarker panel is established, it may then be adapted for routine non-invasive clinical screening using a feasible biospecimen type, such as feces, saliva, or their combination.

### Microbiome-based clinical interventions

In routine clinical oncology, the tumor microbiome remains largely overlooked. While research in this area is actively ongoing and accumulating evidence points to the role of the CRC microbiome in treatment response, translation into clinical practice remains limited and is still in its early stages (Incognito et al., 2025; Parajuli et al., 2025). Conceptualizing CRC-associated communities as structured units operating according to defined rules opens a path toward precision therapy (Choudhury et al., 2025) and offers an alternative to antibiotics, which further promote dysbiosis (Kabwe et al., 2026; Wuyi and Tao, 2026).

Targeted interruption of physical and metabolic interactions within the consortium, such as blocking the bridging, adhesion, colonization, or mediating functions of key organisms, has the potential to destabilize and collapse the biofilm *in vitro* (Kaplan et al., 2009; Liu et al., 2010; Abed et al., 2016; Casasanta et al., 2020; Choudhury et al., 2025; Incognito et al., 2025; Wuyi and Tao, 2026). Similarly, inhibition of virulence factor production or their direct neutralization may reduce the oncogenic potential of the microbiota without untargeted bacterial elimination (as shown in *in vitro* experiments) (Rubinstein et al., 2013; Casasanta et al., 2020; Meng et al., 2021; Wuyi and Tao, 2026). While such approaches are discussed as viable therapeutic options in recent clinical profiling studies (Parajuli et al., 2025) and comprehensive reviews (Chen et al., 2022a; Wuyi and Tao, 2026), evidence for targeted anti-virulence strategies currently remains at the preclinical proof-of-concept stage. Nevertheless, the successful FDA approval of monoclonal antibodies against *Cl. difficile* toxins establishes a promising clinical precedent for the translation of targeted gut anti-virulence approaches (Secore et al., 2017; Gerding et al., 2018).

Highly specific tools are currently under development. These include monoclonal antibodies, small-molecule inhibitors (supported by comprehensive reviews of preclinical *in vitro* and animal models) (Incognito et al., 2025; Wuyi and Tao, 2026), selective phage therapy (demonstrated in *in vitro* cell line assays and *in vivo* animal models) (Zheng et al., 2019; Incognito et al., 2025; Kabwe et al., 2026; Wuyi and Tao, 2026), and genetically engineered probiotics (validated in *in vitro* polymicrobial community models) (Choudhury et al., 2025). A 16S rDNA sequencing-based analysis showed that such approaches are associated with reduced local immunosuppression and substantially enhanced sensitivity of cancer cells to immuno- and chemotherapy (Parajuli et al., 2025). However, microbiome-directed therapeutics remain at an early preclinical stage. Strategies targeting bridging functions, adhesion, virulence-factor activity, or biofilm stability require staged validation of efficacy, safety, resistance risk, microbiome-wide ecological effects, and compatibility with standard anticancer therapy before clinical translation can be considered. Ultimately, the integration of microbiome-directed principles represents a critical next step in oncology (Incognito et al., 2025; Liu et al., 2025; Kabwe et al., 2026; Wuyi and Tao, 2026).

## Conclusion

In this work, we propose an expanded perspective on the oral consortium and its key members (members of the genera *Fusobacterium, Streptococcus, Campylobacter*, species *Po. gingivalis, Pr. intermedia, Pa. micra, Ps. stomatis*, and *Cn. albicans*), whose structural-ecological properties and functional characteristics are associated with facilitated translocation, survival and promotion of primary dysbiotic and carcinogenic disruptions in the intestine. Oral bacteria do not colonize tumors randomly, but translocate via enteral or hematogenous routes as intact communities and reconstitute analogous polymicrobial consortia, incorporating intestinal species in the process. The success of colonization and the maintenance of chronic inflammation within CRC consortia are likely facilitated by the active participation of established and putative bridge organisms. While these taxa facilitate synergistic cooperation of virulence factors across the community, substantially amplifying overall pathogenic potential, their structural scaffolding capacity within the CRC microenvironment requires direct spatial validation. Within the original framework of the driver-passenger model, oral bacteria were classified merely as opportunistic passengers. However, with the ongoing discovery and characterization of their specific virulence factors, it is now more accurate to reclassify many of these pathobionts as potential CRC enhancers, as suggested by preclinical models. Consequently, the role of specific taxa in tumor progression should be defined by the functional activity and ecological impact of their virulence determinants. The compilation includes structural characteristics of each determinant, its localization, and primary pathogenic function, providing a convenient foundation for further investigation of their polymicrobial synergy in CRC initiation and progression. The structured polymicrobial communities and their internal interactions are epidemiologically associated with the clinical trajectory of CRC. The ecological stability and virulence enhancement provided by bridge species and community synergists strongly correlate with measurable outcomes for patients: poor prognosis, chemoresistance, and disease progression. Consequently, these insights suggest that actively disrupting the synergistic interactions and structural integrity of the entire carcinogenic consortium represents a rational and promising strategy for precision CRC treatments.
