# Supplementary material for: Oral microbiome contribution in colorectal carcinogenesis: polymicrobial interactions and virulence determinants
Source: Front Cell Infect Microbiol. 2026 Jul 28;16:1887826. doi: 10.3389/fcimb.2026.1887826 (PMC13457010; doi:10.3389/fcimb.2026.1887826)
Supplement: Supplementary file 1 [file DataSheet1.pdf]

Supplementary Material

Supplementary Table 1. Evidence-based classification of CRC-associated oral taxa according to proposed oral-to-gut translocation pathways and current levels of evidence. The table summarizes key CRC-associated oral microorganisms and evaluates the evidence supporting their potential dissemination from the oral cavity to the gut in CRC context. For each taxon, the table details the reported experimental or clinical findings on translocation, the proposed anatomical route (enteral and hematogenous, or not yet established), the corresponding confidence level, and its relevance within the CRC context. The summarized taxa include *F. animalis*, the *F. nucleatum* species complex, *S. odontolytica*, *Streptococcus* spp., *P. gingivalis*, *P. micra*, and *P. anaerobius*. The data highlight that CRC-associated oral taxa may reach or persist in the gut through distinct routes, contributing to colorectal carcinogenesis through tumor colonization, epithelial adhesion and invasion, inflammatory signaling, immune modulation, biofilm formation, and activation of pro-tumorigenic pathways.

To assess current evidence for oral bacterial translocation to the gut and their role in colorectal cancer (CRC) progression, we ranked the evidence by strength of supporting data (see Supplementary Table 1). **Level 1** (Highest): in vivo human tissue tracing and spatial evidence. For translocation and colonic colonization, this relies on the isolation of identical clonal strains from both the oral cavity and CRC tumors in the same patient (e.g., via strain-level whole-genome sequencing). For a causal role in CRC progression, highest-level evidence requires targeted removal of a high-risk strain in carriers demonstrating reduced recurrence of advanced adenomas or other validated endpoints; or the detection of specific bacteria-induced molecular alterations in patient tumor cells in situ, for example, specific mutations or epigenetic "signatures"; or prospective detection of a specific strain or virulence factor years before diagnosis with controlled confounders and a clear dose-response effect, or longitudinal observation showing that an adenoma harboring a specific taxon or virulome has a higher risk of accelerated growth, high-grade dysplasia, or transition to carcinoma compared to adenomas without it. **Level 2:** animal models of CRC (e.g., oral gavage of pathogens resulting in specific colon tumor colonization and accelerated tumorigenesis). **Level 3:** in vitro experiments (e.g., cell culture assays, oral infection/periodontitis models). These studies prove the biological capacity for translocation (such as intracellular survival or co-aggregation) and potential pro-tumorigenic properties, but require extrapolation to CRC in vivo. **Level 4 (Lowest)**: sequence-based clinical correlations (e.g., 16S rRNA/metagenomic co-occurrence in fecal or tumor samples), with direct spatial visualization of physical bacterial interactions in human tumor biofilms (e.g., using fluorescence in situ hybridization), as a more advanced variant of Level 4. While confirming the presence of oral taxa in the gut and their disease association, these methods cannot independently verify the translocation route or their causal role in tumor development.

Key taxa	Translocation Pathway Evidence	Evidence in CRC context
Fusobacterium animalis	**Enteral**<br>***In vivo*, level 2 (supporting *in vitro*, level 3):** oral gavage of representative *F. animalis* strains in DSS-treated *Apc* Min+/− mice significantly increased large-intestinal adenoma burden compared with vehicle controls, supporting enteral exposure and CRC-promoting activity (1,2). One mechanism that may facilitate oral-to-gut passage is the glutamate-dependent acid resistance (GDAR) system, which enables *F. animalis* strains to survive simulated extreme gastric acidity (pH 3) (2).	***In vivo*, level 2 (supporting *in vitro*, level 3):** ApcMin+/− mouse model with induced colitis. Linked to CRC progression through tumor niche adaptation, metabolic remodeling, and proinflammatory immune modulation. Single oral gavage increased intestinal adenoma burden and altered glutathione, oxidative stress, and inflammatory lipid pathways (2). Earlier enteral feeding experiments showed increased tumor multiplicity in *Apc*^Min/+ mice, with intratumoral myeloid recruitment and an NF-κB-related proinflammatory signature shared with human Fusobacterium positive CRC (1). In human CRC tissues, subsequent molecular and spatial biofilm analysis showed that *F. animalis* is enriched in biofilm-positive colon tumors, supporting a structured tumor-associated biofilm niche (3).

Key taxa	Translocation Pathway Evidence	Evidence in CRC context
***Fusobacterium nucleatum* species complex**	**Enteral**<br>***In vivo*, level 2:** evidence is supported by experimental oral administration models, in which enterally delivered *F. nucleatum* was subsequently detected and spatially localized across multiple intestinal compartments, including stool, intestinal mucosa, submucosal regions, tumor tissue, and colonic crypts, using complementary methodologies such as qPCR, FISH/IF, and *in situ* hybridization. These findings are corroborated by human data demonstrating the presence and enrichment of *F. nucleatum* in stool specimens, colorectal adenomas, and carcinoma tissues, as well as its spatial organization into discrete microcolonies within human colonic crypts (1,4,5).<br>**Oral-gut translocation**<br>***In vivo*, level 1:** Paired saliva and colorectal cancer tissue samples from CRC patients were cultured on Fusobacterium-selective agar, and colonies were screened by *F. nucleatum*-specific PCR. Subspecies were identified by 16S rRNA gene sequencing, and matched CRC–saliva isolates were compared by AP-PCR strain typing. Identical AP-PCR fingerprint patterns were detected in saliva and CRC tissue from the same patients, supporting oral-to-CRC translocation of the *F. nucleatum* species complex (6).<br><br>**Hematogenous**<br>***In vivo*, level 2:** intravenous (IV) injection of *F. nucleatum* in orthotopic murine CRC models results in highly efficient, preferential colonization of colon tumors (far exceeding oral gavage efficiency). This specific tumor tropism is mediated by the bacterial Fap2 lectin binding to host Gal-GalNAc overexpressed on CRC cells (7,8).	***In vivo*, level 2 (supporting *in vitro*, level 3):** in *Apc*^Min/+ mice, *F. nucleatum* increased intestinal tumor multiplicity and induced a pro-inflammatory tumor microenvironment through selective recruitment of tumor-infiltrating myeloid cells (1). *F. nucleatum* also enhanced CRC cell proliferation, invasion, xenograft tumor formation, and tumor development in mice by activating TLR4–MYD88–NF-κB signaling, leading to miR-21 upregulation and RASA1 suppression (4). The IV injection study further supports the biological relevance of this enrichment by showing that hematogenous delivery enables highly efficient colon tumor colonization (7).<br>*F. nucleatum* was shown to colonize deep gut crypts, bind LY6A+ revival stem cells through LY6A, induce RPS14 upregulation, and promote hyperproliferation and tumorigenic transformation of these cells *in vitro* (5). Also *F. nucleatum* is described as a CRC-associated bacterium capable of promoting colonic tumor formation in preclinical models, while the Fap2–Gal-GalNAc interaction explains how *F. nucleatum* becomes selectively enriched in CRC tissue (8).
Schaalia odontolytica (Actinomyces odontolyticus)	**Enteral**<br>**Sequencing, level 4:** Paired oral–fecal analysis identified an ASV mapped to *S. odontolytica* that was shared between oral and stool samples and was significantly more abundant in oral than stool samples (9). A large strain-resolved metagenomic study further identified *S. odontolytica* as a highly transmissible oral species, while showing that oral–fecal overlap across healthy individuals is often driven by same-strain oral-to-gut transmission (10).	***In vivo*, level 2 (supporting sequencing, level 4):** *S. odontolytica* is linked to early colorectal carcinogenesis by both clinical association and mechanistic evidence (11). Fecal metagenomic analysis showed that *S. odontolytica* was significantly increased in multiple polypoid adenomas and/or intramucosal carcinomas. Vesicles from bacteria activate NF-κB and TLR2-mediated inflammatory signaling, enter colonic epithelial cells, co-localize with mitochondria, induce mitochondrial dysfunction, ROS production, DNA damage, and colonic dysplasia in mice (12).
***Streptococcus* spp.**	**Enteral**<br>***In vivo*, level 2:** Oral gavage of *S. gallolyticus* subsp. *gallolyticus* TX20005 in mouse CRC models led to detectable gut/colon colonization, confirmed by fecal qPCR and immunofluorescent detection in colonic/tumor tissues after gavage (13,14).	***In vivo*, levels 2-4:** S. *gallolyticus* subsp. *gallolyticus* stimulates proliferation of CRC cell lines in a model-dependent manner, requiring viable bacteria and direct bacteria–host contact, with concomitant upregulation of β-catenin, c-Myc, cyclin D1, and PCNA (13). In AOM-induced murine CRC models, its exposure increased tumor burden, dysplasia grade, colonic epithelial proliferation, and β-catenin staining (13).

Key taxa	Translocation Pathway Evidence	Evidence in CRC context
	In vivo*, level 2:** *S. bovis* was administered by oral gavage to AOM/DSS-treated mice, and DNA-based qPCR quantification was used to verify increased fecal *S. bovis* abundance after pretreatment (15).<br>**Sequencing/metatranscriptomics, level 4:** human stool RNA analysis demonstrates that *Streptococcus* oral taxa do not merely pass through the GI tract as dead DNA, but remain alive, metabolically active, and upregulate virulence/antibiotic resistance genes in the gut (16). Additional evidence of an oral–gut association comes from paired saliva–stool 16S rRNA gene profiling, which identified oral-associated *Streptococcus* taxa enriched in CRC patients (17).<br><br>**Hematogenous**<br>In vitro*, level 3:** oral *Streptococcus* spp. can invade and survive within circulating peripheral blood mononuclear cells (PBMCs) and dendritic cells in patients with periodontitis and also induce rapid monocyte-to-mature DC differentiation (18,19).	Subsequently, the T7SS^T05 type VII secretion system was identified as a key virulence determinant required for adherence to CRC cells, β-catenin/PCNA upregulation, colonic colonization, and tumor promotion (14).<br>***In vivo*, level 2 (supporting *in vitro*, level 3):** *S. bovis* promotes CRC tumorigenesis in AOM/DSS mice by increasing tumor number and size and by inducing a pro-tumor inflammatory immune microenvironment characterized by elevated IL-6, Scyb1/IL-8-like chemokine, Ptgs2/COX-2, IL-1β, TNF, and Ccl2, together with recruitment of tumor-infiltrating CD11b+TLR-4+ myeloid cells (15).<br>**Sequencing, level 4:** gene profiling identified *Streptococcus* taxa with significantly higher relative abundance in both saliva and stool samples from CRC patients compared with healthy controls, supporting an association between these taxa and CRC-related oral–gut dysbiosis (17). Several *Streptococcus* species were transcriptionally active in the CRC gut environment and showed increased transcriptional CRC-regulated activity related to host colonization, virulence, antibiotic resistance, acid/stress adaptation, and altered oxidative-stress responses. These findings indicate microbial activity and adaptation in the CRC gut (16).
Porphyromonas gingivalis	**Enteral**<br>***In vivo*, level 2:** oral gavage of *P. gingivalis* in chemically induced (AOM/DSS) murine models effectively translocates to the gut as shown by 16S rRNA gene sequencing of lumen-associated microbiota (LAM) and mucosa-associated microbiota (MAM) and strongly adheres to the intestinal epithelium, as confirmed by Caco-2 adhesion assays (20). In another model, orally administered *P. gingivalis* was detected in colon tumor tissue by IHC, FISH, and qPCR (21).<br><br>**Hematogenous**<br>***In vitro*, level 3:** in periodontitis, *P. gingivalis* is enriched in oral biofilms and shows increased expression of the *mfa-1*, which promotes uptake by dendritic cells, can invade and survive within circulating peripheral blood mononuclear cells (PBMCs) and dendritic cells (DCs); it is detected in circulating blood DCs, its abundance in oral biofilm correlates with its abundance in DCs (18,22).	***In vivo*, level 2:** Exposure to *P. gingivalis* increases tumor burden and aggravates colitis-associated CRC in mice, upregulating the expression of CRC markers including β-catenin and PCNA (20). Mechanistically, the reported effects included activation of the hematopoietic NLRP3 inflammasome, recruitment of myeloid cells, formation of a proinflammatory tumor microenvironment (21), mucosal enrichment mediated by gingipain-dependent adhesion to intestinal epithelial cells (20), and CHI3L1-mediated impairment of iNKT-cell cytotoxicity (23).
Parvimonas micra	**Enteral**<br>***In vivo*, level 2:** oral gavage of CRC-derived *P. micra* strains into *ApcMin/+* mice successfully translocates to the gut. After resident microbiota depletion, the mice were gavaged with *P. micra* with subsequent detection of increased fecal abundance. In germ-free mice, intestinal colonization was further confirmed by FISH (24).	***In vivo*, level 2:** promotes intestinal tumor formation in mice by upregulating proliferative pathways: 1) increased colonic epithelial cell proliferation: Ki-67 and PCNA; 2) increased expression of genes involved in angiogenesis, cell proliferation, invasion, and metastasis; 3) down-regulation of genes involved in DNA repair and apoptosis; 4) soluble factors in *P. micra* conditioned media induced HT29 cell proliferation and increased activated β-catenin, cyclin D1, and c-myc, suggesting

Key taxa	Translocation Pathway Evidence	Evidence in CRC context
	**Hematogenous**<br>***In vitro*, level 3:** *P. micra* has a proven ability to thrive in human serum and establish extraoral infections strongly supports transient bacteremia as a highly viable route for its hematogenous dissemination to the gut (25,26)<br><br>**Oral-gut translocation**<br>***In vivo*, level 1:** genomic comparison identified highly similar isolates from subgingival crevicular fluid and colorectal adenocarcinoma in the same patient. The paired PM89KC isolates showed >99% nucleotide identity, conserved genome organization, shared CRISPR-Cas features, and duplicated prophages, supporting a recent common ancestor and translocation from the oral cavity to CRC tissue (27).	Wnt/β-catenin activation; 5) inflammatory/immune mechanism: upregulated chemotaxis-related genes, Il-17a, Il-22, Il-23a, Th17 infiltration, IL-17 staining, and differentiation of $CD4^+$ T cells into $IL\text{-}17^+CD4^+$ phenotype (24).<br>**Sequencing, level 4:** genomic and metatranscriptomic analyses showed that *P. micra* was active in colorectal adenocarcinoma and expressed predicted virulence-associated factors, including ABC transporters, type IV secretion components such as PcfB and PrgI, NlpC/P60 and MltA cell-wall remodeling proteins, tissue adhesins, peptidases, neutrophil-activating proteins, and biofilm-related factors (27).
Peptostreptococcus anaerobius	**Enteral**<br>***In vivo*, level 2:** oral gavage of *P. anaerobius* strains into *ApcMin/+* mice translocates to the gut. After gavage, gut/colon colonization was inferred from species-level qPCR detection in stool and FISH visualization in colon/tumor tissue; CRC-bearing mice showed higher stool abundance and stronger colonic colonization than naive mice (28). In addition, mice were pretreated with antibiotics to deplete resident microbiota prior to gavage, which enhanced gut colonization efficiency (29)	***In vivo*, level 2:** CRC-derived *P. anaerobius* strains (*P. anaerobius* strain ATCC 27337 (29)) in gut *ApcMin/+* mice promote intestinal tumor formation by upregulating proliferative pathways. PCWBR2-α2/β1 complex activates FAK–PI3K–Akt and NF-κB. *P. anaerobius* also remodels the tumor immune microenvironment by expanding MDSCs and TANs (28). Subsequent work conducted in mice further supported an integrin α2β1–NF-κB–CXCL1 axis that recruits CXCR2+ MDSCs and suppresses functional antitumor T-cell responses (29).

References

1. Kostic AD, Chun E, Robertson L, Glickman JN, Gallini CA, Michaud M, et al. Fusobacterium nucleatum Potentiates Intestinal Tumorigenesis and Modulates the Tumor-Immune Microenvironment. Cell Host Microbe. 2013 Aug;14(2):207–15. doi:10.1016/j.chom.2013.07.007

2. Zepeda-Rivera M, Minot SS, Bouzek H, Wu H, Blanco-Míguez A, Manghi P, et al. A distinct *Fusobacterium nucleatum* clade dominates the colorectal cancer niche. Nature. 2024 Apr 11;628(8007):424–32. doi:10.1038/s41586-024-07182-w

3. Queen J, Cing Z, Minsky H, Nandi A, Southward T, Ferri J, et al. Fusobacterium nucleatum is enriched in invasive biofilms in colorectal cancer. Npj Biofilms Microbiomes. 2025 May 20;11(1):81. doi:10.1038/s41522-025-00717-7

4. Yang Y, Weng W, Peng J, Hong L, Yang L, Toiyama Y, et al. Fusobacterium nucleatum Increases Proliferation of Colorectal Cancer Cells and Tumor Development in Mice by Activating Toll-Like Receptor 4 Signaling to Nuclear Factor−κB, and Up-regulating Expression of MicroRNA-21. Gastroenterology. 2017 Mar;152(4):851-866.e24. doi:10.1053/j.gastro.2016.11.018

5. Wang Q, Hu T, Zhang Q, Zhang Y, Dong X, Jin Y, et al. Fusobacterium nucleatum promotes colorectal cancer through neogenesis of tumor stem cells. J Clin Invest. 2025 Feb 3;135(3):e181595. doi:10.1172/JCI181595

6. Komiya Y, Shimomura Y, Higurashi T, Sugi Y, Arimoto J, Umezawa S, et al. Patients with colorectal cancer have identical strains of *Fusobacterium nucleatum* in their colorectal cancer and oral cavity. Gut. 2019 Jul;68(7):1335–7. doi:10.1136/gutjnl-2018-316661

7. Abed J, Maalouf N, Manson AL, Earl AM, Parhi L, Emgård JEM, et al. Colon Cancer-Associated Fusobacterium nucleatum May Originate From the Oral Cavity and Reach Colon Tumors via the Circulatory System. Front Cell Infect Microbiol. 2020 Aug 7;10:400. doi:10.3389/fcimb.2020.00400

8. Abed J, Emgård JEM, Zamir G, Faroja M, Almogy G, Grenov A, et al. Fap2 Mediates Fusobacterium nucleatum Colorectal Adenocarcinoma Enrichment by Binding to Tumor-Expressed Gal-GalNAc. Cell Host Microbe. 2016 Aug;20(2):215–25. doi:10.1016/j.chom.2016.07.006

9. Rashidi A, Koyama M, Dey N, McLean JS, Hill GR. Colonization resistance is dispensable for segregation of oral and gut microbiota. BMC Med Genomics. 2023 Feb 22;16(1):31. doi:10.1186/s12920-023-01449-3

10. Heidrich V, Fackelmann G, Ricci L, Spadazzi R, Baldanzi G, Punčochář M, et al. Strain transmission links human microbiomes along the oral-gut axis and across cohabiting individuals. Cell Press Blue. 2026 Jun;1(3):100034. doi:10.1016/j.cpblue.2026.100034

11. Yachida S, Mizutani S, Shiroma H, Shiba S, Nakajima T, Sakamoto T, et al. Metagenomic and metabolomic analyses reveal distinct stage-specific phenotypes of the gut microbiota in colorectal cancer. Nat Med. 2019 Jun 6;25(6):968–76. doi:10.1038/s41591-019-0458-7

12. Miyakawa Y, Otsuka M, Shibata C, Seimiya T, Yamamoto K, Ishibashi R, et al. Gut Bacteria-derived Membrane Vesicles Induce Colonic Dysplasia by Inducing DNA Damage in Colon Epithelial Cells. Cell Mol Gastroenterol Hepatol. 2024;17(5):745–67. doi:10.1016/j.jcmgh.2024.01.010

13. Kumar R, Herold JL, Schady D, Davis J, Kopetz S, Martinez-Moczygemba M, et al. Streptococcus gallolyticus subsp. gallolyticus promotes colorectal tumor development. Sullam PM, editor. PLOS Pathog. 2017 Jul 13;13(7):e1006440. doi:10.1371/journal.ppat.1006440

14. Taylor JC, Gao X, Xu J, Holder M, Petrosino J, Kumar R, et al. A type VII secretion system of Streptococcus gallolyticus subsp. gallolyticus contributes to gut colonization and the development of colon tumors. Prince A, editor. PLOS Pathog. 2021 Jan 6;17(1):e1009182. doi:10.1371/journal.ppat.1009182

15. Deng Q, Wang C, Yu K, Wang Y, Yang Q, Zhang J, et al. Streptococcus bovis Contributes to the Development of Colorectal Cancer via Recruiting $CD11b^{+}TLR\text{-}4^{+}$ Cells. Med Sci Monit. 2020 Jun 4;26. doi:10.12659/MSM.921886

16. Lamaudière MTF, Arasaradnam R, Weedall GD, Morozov IY. The Colorectal Cancer Gut Environment Regulates Activity of the Microbiome and Promotes the Multidrug Resistant Phenotype of ESKAPE and Other Pathogens. Young VB, editor. mSphere. 2023 Apr 20;8(2):e00626-22. doi:10.1128/msphere.00626-22

17. Uchino Y, Goto Y, Konishi Y, Tanabe K, Toda H, Wada M, et al. Colorectal Cancer Patients Have Four Specific Bacterial Species in Oral and Gut Microbiota in Common—A Metagenomic Comparison with Healthy Subjects. Cancers. 2021 Jul 2;13(13):3332. doi:10.3390/cancers13133332

18. El-Awady A, De Sousa Rabelo M, Meghil MM, Rajendran M, Elashiry M, Stadler AF, et al. Polymicrobial synergy within oral biofilm promotes invasion of dendritic cells and survival of consortia members. Npj Biofilms Microbiomes. 2019 Mar 18;5(1):11. doi:10.1038/s41522-019-0084-7

19. Hahn CL, Schenkein HA, Tew JG. Endocarditis-Associated Oral Streptococci Promote Rapid Differentiation of Monocytes into Mature Dendritic Cells. Infect Immun. 2005 Aug;73(8):5015–21. doi:10.1128/IAI.73.8.5015-5021.2005

20. Motosugi S, Takahashi N, Mineo S, Sato K, Tsuzuno T, Aoki-Nonaka Y, et al. Enrichment of Porphyromonas gingivalis in colonic mucosa-associated microbiota and its enhanced adhesion to epithelium in colorectal carcinogenesis: Insights from in vivo and clinical studies. PLOS ONE. 2025 Mar 25;20(3):e0320383. doi:10.1371/journal.pone.0320383

21. Wang X, Jia Y, Wen L, Mu W, Wu X, Liu T, et al. *Porphyromonas gingivalis* Promotes Colorectal Carcinoma by Activating the Hematopoietic *NLRP3* Inflammasome. Cancer Res. 2021 May 1;81(10):2745–59. doi:10.1158/0008-5472.CAN-20-3827

22. Carrion J, Scisci E, Miles B, Sabino GJ, Zeituni AE, Gu Y, et al. Microbial Carriage State of Peripheral Blood Dendritic Cells (DCs) in Chronic Periodontitis Influences DC Differentiation, Atherogenic Potential. J Immunol. 2012 Sep 15;189(6):3178–87. doi:10.4049/jimmunol.1201053

23. Díaz-Basabe A, Lattanzi G, Perillo F, Amoroso C, Baeri A, Farini A, et al. *Porphyromonas gingivalis* fuels colorectal cancer through CHI3L1-mediated iNKT cell-driven immune evasion. Gut Microbes. 2024 Dec 31;16(1):2388801. doi:10.1080/19490976.2024.2388801

24. Zhao L, Zhang X, Zhou Y, Fu K, Lau HCH, Chun TWY, et al. Parvimonas micra promotes colorectal tumorigenesis and is associated with prognosis of colorectal cancer patients. Oncogene. 2022 Sep 2;41(36):4200–10. doi:10.1038/s41388-022-02395-7

25. Higashi DL, Krieger MC, Qin H, Zou Z, Palmer EA, Kreth J, et al. Who is in the driver's seat? *Parvimonas micra* : An understudied pathobiont at the crossroads of dysbiotic disease and cancer. Environ Microbiol Rep. 2023 Aug;15(4):254–64. doi:10.1111/1758-2229.13153

26. Watanabe T, Hara Y, Yoshimi Y, Fujita Y, Yokoe M, Noguchi Y. Clinical characteristics of bloodstream infection by Parvimonas micra: retrospective case series and literature review. BMC Infect Dis. 2020 Dec;20(1):578. doi:10.1186/s12879-020-05305-y

27. Conde-Pérez K, Buetas E, Aja-Macaya P, Martin-De Arribas E, Iglesias-Corrás I, Trigo-Tasende N, et al. *Parvimonas micra* can translocate from the subgingival sulcus of the human oral cavity to colorectal adenocarcinoma. Mol Oncol. 2024 May;18(5):1143–73. doi:10.1002/1878-0261.13506

28. Long X, Wong CC, Tong L, Chu ESH, Ho Szeto C, Go MYY, et al. Peptostreptococcus anaerobius promotes colorectal carcinogenesis and modulates tumour immunity. Nat Microbiol. 2019 Sep 9;4(12):2319–30. doi:10.1038/s41564-019-0541-3

29. Liu Y, Wong CC, Ding Y, Gao M, Wen J, Lau HCH, et al. Peptostreptococcus anaerobius mediates anti-PD1 therapy resistance and exacerbates colorectal cancer via myeloid-derived suppressor cells in mice. Nat Microbiol. 2024 May 15;9(6):1467–82. doi:10.1038/s41564-024-01695-w
