# Supplementary material for: Oral microbiome contribution in colorectal carcinogenesis: polymicrobial interactions and virulence determinants
Source: Front Cell Infect Microbiol. 2026 Jul 28;16:1887826. doi: 10.3389/fcimb.2026.1887826 (PMC13457010; doi:10.3389/fcimb.2026.1887826)
Supplement: Supplementary file 2 [file DataSheet2.pdf]

Supplementary Material

Supplementary Table 2. Structural and functional annotation of CRC-associated virulence factors. The table summarizes 14 key virulence factors (VFs) of CRC-associated oral taxa and provides a comprehensive overview of each determinant by detailing its structural or fold type, cellular localization, genomic carrier/location, general functional roles, and corresponding microbial entities. The annotated VFs represent surface-exposed adhesins, autotransporter proteins, fimbrial components, gingipain proteases, and fungal cell-surface factor. These determinants act synergistically to facilitate interbacterial cooperation and biofilm formation, drive tissue invasion and epithelial barrier disruption, and modulate host immune responses.

Expression evidence in CRC niche (levels):

- **E1** – protein detected in human CRC tissue/mucosa
- **E2** – transcript detected in human CRC tissue/mucosa
- **E3** – transcript/protein detected in CRC fecal/luminal samples
- **E4** – transcript/protein detected in CRC experimental models
- **E5** – transcript/protein detected in CRC-derived isolates under relevant *in vitro*/*ex vivo* conditions
- **E6** – expression shown only outside CRC context (oral biofilm, infection model, laboratory strain, etc.)
- **E0** – gene presence only; no expression evidence

VF Short Name	VF Full Name	Structural type (fold type)	Localization	Carrier/ Location	General Role	Expression evidence in CRC niche	Entities
Aid1	Adherence Inducing Determinant 1	α-membrane monomeric lipoprotein	Outer membrane	Chromosome	Interbacterial adhesin, interbacterial co-aggregation and biofilm formation regulator	**E6 level:** Expression has been demonstrated only in *in vitro* biofilm/interbacterial interaction models (1,2)	*F. nucleatum* subsp. *nucleatum* (1,3–5)<br>*F. animalis* (5)<br>*F. vincentii* (5)<br>*F. polymorphum* (5)
Aim1	Apoptosis-inducing membrane protein 1	C-terminal β-barrel + β-helix passenger	Outer membrane	Chromosome	Adhesin	**E2 level:** mRNA-level evidence in human CRC tumor tissue by metatranscriptomic analysis; tumor-expressed *bigA*-annotated *Fusobacterium* transcripts mapped to several *F. nucleatum* T5aSS/autotransporter virulence genes, including Aim1 (6)	*F. animalis* (clade C2 in the article) (7,8)<br>*F. watanabei* (clade C1 in the article) (7)<br>*F. nucleatum* subsp. *nucleatum* (9,10)

VF Short Name	VF Full Name	Structural type (fold type)	Localization	Carrier/ Location	General Role	Expression evidence in CRC niche	Entities
Als3	Agglutinin-like sequence protein 3	Ig-like β-sandwich N-domain, β-strand–rich tandem-repeat stalk, C-terminal GPI-anchor signal	Cell surface	Chromosome	Adhesin, invasin, interbacterial biofilm formation factor, nutrient acquisition system	**E4 level:** an association with intestinal inflammatory processes has been established; in colon contents of CRC mouse models, co-culture with *F. nucleatum* induced upregulation of *ALS3* and other hypha-associated genes (11–14)	*C. albicans* (13)
CmpA	Coaggregation mediating protein A	Monomeric autotransporter adhesin (C-terminal β-barrel, N-terminal β-helix fold)	Outer membrane	Chromosome	Interbacterial adhesin	**E5 level:** *cmpA* was upregulated in *F. animalis* cells under ethanolamine exposure, supporting inducible expression under CRC niche–relevant metabolic conditions; expression was shown in CRC-associated/CRC-derived *Fusobacterium* isolates under relevant *in vitro* conditions (7)	*F. nucleatum* subsp. *nucleatum* (15,16)<br>*F. animalis* (clade C2 in the article) (7)
FadA	Fusobacterium Adhesin A	Filamentous, α-helical coiled-coil with hairpin loop	Outer membrane	Chromosome	Adhesin, invasin, interbacterial co-aggregation regulator	**E1 level:** direct protein-level evidence in human CRC tissue is available: FadA protein was detected at higher levels in CRC tumors than in adjacent normal tissues by immunofluorescence, with co-localization with Annexin A1 in tumors. Additional experimental CRC models (E4) support its functional role in adhesion, invasion, β-catenin signaling, and tumor growth (17–19)	*F. nucleatum* subsp. *nucleatum* (17,18,20)<br>*F. hwasookii* (21,22)<br>*F. varium* (22)<br>*F. necrophorum* (22,23)
FadA2	Fusobacterium Adhesin A homolog	Filamentous, α-helical coiled-coil with hairpin loop	Outer membrane	Chromosome	Adhesin, invasin, interbacterial co-aggregation regulator	**E0 level:** *fadA2* presence varied among CRC-derived and non-CRC *F. nucleatum* strains (5,7,24)	*F. varium* (22,25)<br>*F. ulcerans* (22,26)<br>*F. animalis* (clade C2 in the article) (7,8)<br>*F. watanabei* (clade C1 in the article) (7,8)
FadA3	Fusobacterium Adhesin A homolog	Filamentous, α-helical coiled-coil with hairpin loop	Outer membrane	Chromosome	Adhesin, invasin, interbacterial co-aggregation regulator (dosage effect)	**E0 level:** *fadA3* was detected as a FadA-family homologue, including CRC-derived strains, by whole-genome sequencing and comparative genomic analysis and was universally present, with multiple copies in several *Fusobacterium* strains (5,24,27)	*F. ulcerans* (28)<br>*F. varium* (25,28)

<table>
<tr><th>VF Short Name</th><th>VF Full Name</th><th>Structural type (fold type)</th><th>Localization</th><th>Carrier/ Location</th><th>General Role</th><th>Expression evidence in CRC niche</th><th>Entities</th></tr>
<tr><td rowspan="2">Fap2</td><td rowspan="2">Fusobacterium autotransporter protein 2</td><td rowspan="2">Autotransporter adhesin (β-barrel at C-terminus, β-helix/lectin-like passenger domain at N-terminus)</td><td rowspan="2">Outer membrane</td><td rowspan="2">Chromosome</td><td rowspan="2">Adhesin, invasin, interbacterial co-aggregation regulator, hemagglutination factor</td><td rowspan="6">**E2 level:** metatranscriptomic data show tumor-enriched expression of *F. nucleatum*-derived virulence genes corresponding to Fap2/RadD and related T5aSS autotransporters (6)</td><td>*F. nucleatum* subsp. *nucleatum* (29–31)</td></tr>
<tr><td>*F. animalis* (clade C2 in the article) (7,8)</td></tr>
<tr><td rowspan="4">RadD</td><td rowspan="4">Arginine-inhibited membrane protein</td><td rowspan="4">β-barrel autotransporter (T5SS fold)</td><td rowspan="4">Outer membrane</td><td rowspan="4">Chromosome</td><td rowspan="4">Adhesion, polymicrobial co-aggregation factor</td><td>*F. animalis* (clade C2 in the article) (7,8)</td></tr>
<tr><td>*F. watanabei* (clade C1 in the article) (7,8)</td></tr>
<tr><td>*F. nucleatum* subsp. *nucleatum* (7,30,32)</td></tr>
<tr><td>*F. polymorphum* (2,33,34)</td></tr>
<tr><td>FimA</td><td>FimA LONG/ MAJOR fimbriae genotype Ib</td><td>Transthyretin-like β-sandwich (Greek-key)</td><td>Outer membrane</td><td>Chromosome</td><td>Adhesin, invasin and biofilm formation factor</td><td>**E0/E6 levels:** *P. gingivalis* showed mucosal enrichment in CRC-related intestinal samples and strain-dependent fimbriae-associated adhesion to intestinal/CRC epithelial cells; however, direct FimA or *fimA* genotype Ib expression in the CRC niche was not demonstrated. (35,36)</td><td>*P. gingivalis* (35,37)</td></tr>
<tr><td>Kgp</td><td>Lysine-specific gingipain</td><td>Multidomain cysteine endopeptidase, catalytic domain with caspase-like fold</td><td>Outer membrane, outer membrane vesicles</td><td>Chromosome</td><td>Protease, adhesin-like factor, biofilm suppressive and regulatory factor, kinin-system–modulating factor, heme/iron acquisition system, hemagglutination factor</td><td rowspan="2">**E4 level:** *P. gingivalis* secreted factors, including Lys-gingipain/Kgp, were shown to induce EMT-related changes in CRC neoplasia models (38)</td><td rowspan="2">*P. gingivalis* (35)</td></tr>
<tr><td>RgpA</td><td>Arginine-specific gingipain A</td><td>Multidomain cysteine endopeptidase, catalytic domain with caspase-like fold</td><td>Outer membrane, outer membrane vesicles</td><td>Chromosome</td><td>Protease, adhesin-like factor, biofilm-related factor, protein processing factor, heme/iron acquisition system, kinin-system–modulating factor, hemagglutination factor</td></tr>
</table>

VF Short Name	VF Full Name	Structural type (fold type)	Localization	Carrier/ Location	General Role	Expression evidence in CRC niche	Entities
RgpB	Arginine-specific gingipain B	Single-chain cysteine endopeptidase with caspase-like fold	Outer membrane, outer membrane vesicles	Chromosome	Protease, protein processing factor, kinin-system–modulating factor	**E4 level:** has protein-level evidence in a CRC experimental cell model: anti-RgpB immunostaining was used to detect *P. gingivalis* in infected CRC cells, and a gingipain-deficient Δ*rgpA*Δ*rgpB*Δ*kgp* mutant showed reduced ability to promote CRC-cell proliferation and MAPK/ERK activation. (35,36,38)	*P. gingivalis* (35)
PCWBR2	Putative cell wall binding repeat protein 2	α/β-sandwich S-layer protein fold (S-layer-like surface domain)	Cell wall	Chromosome	Adhesin	**E4 level:** has been experimentally identified as a surface protein that directly interacts with integrin α2/β1 on CRC/colonic epithelial cell lines and activates the PI3K–Akt–NF-κB axis in vitro; the tumor-promoting relevance of this pathway was further supported in Apc^Min/+ mouse CRC models (39)	*P. stomatis* (40)

References

1. Kaplan A, Kaplan CW, He X, McHardy I, Shi W, Lux R. Characterization of aid1, a Novel Gene Involved in *Fusobacterium nucleatum* Interspecies Interactions. Microb Ecol. 2014 Aug;68(2):379–87. doi:10.1007/s00248-014-0400-y

2. Shokeen B, Park J, Duong E, Rambhia S, Paul M, Weinberg A, et al. Role of FAD-I in Fusobacterial Interspecies Interaction and Biofilm Formation. Microorganisms. 2020 Jan 2;8(1):70. doi:10.3390/microorganisms8010070

3. Peluso EA, Scheible M, Ton-That H, Wu C. Genetic Manipulation and Virulence Assessment of *Fusobacterium nucleatum*. Curr Protoc Microbiol. 2020 Jun;57(1):e104. doi:10.1002/cpmc.104

4. Song B, Xian W, Sun Y, Gou L, Guo Q, Zhou X, et al. *Akkermansia muciniphila* inhibited the periodontitis caused by Fusobacterium nucleatum. Npj Biofilms Microbiomes. 2023 Jul 17;9(1):49. doi:10.1038/s41522-023-00417-0

5. Queen J, Domingue JC, White JR, Stevens C, Udayasuryan B, Nguyen TTD, et al. Comparative Analysis of Colon Cancer-Derived *Fusobacterium nucleatum* Subspecies: Inflammation and Colon Tumorigenesis in Murine Models. mBio. 2022 Feb 22;13(1):e02991-21. doi:10.1128/mbio.02991-21

6. Buetas E, Conde-Pérez K, Concha Á, Celeiro C, Noguera JF, Bou G, et al. Metatranscriptomic analysis of the microbiota of tumor tissue in colon cancer. Microbiome. 2026 May 12;14(1):155. doi:10.1186/s40168-026-02372-2

7. Zepeda-Rivera M, Minot SS, Bouzek H, Wu H, Blanco-Míguez A, Manghi P, et al. A distinct *Fusobacterium nucleatum* clade dominates the colorectal cancer niche. Nature. 2024 Apr 11;628(8007):424–32. doi:10.1038/s41586-024-07182-w

8. Sivertsen A, Forni D, Molteni C, Bivand J, Dimmen G, Bruvold TS, et al. Reassessing taxonomy and virulence in the *Fusobacterium nucleatum* group—rebuttal of *Fusobacterium animalis* clades " *Fna* C1" and " *Fna* C2," genome announcement for *Fusobacterium watanabei,* and description of *Fusobacterium paranimalis* sp. nov. Han Y, editor. mBio. 2025 Sep 10;16(9):e00941-25. doi:10.1128/mbio.00941-25

9. Kaplan CW, Lux R, Huynh T, Jewett A, Shi W, Haake SK. *Fusobacterium nucleatum* Apoptosis-inducing Outer Membrane Protein. J Dent Res. 2005 Aug;84(8):700–4. doi:10.1177/154405910508400803

10. Zhang X, Wang Y, Fan R, Zhang L, Li Z, Zhang Y, et al. Quantitative Proteomic Analysis of Outer Membrane Vesicles from *Fusobacterium nucleatum* Cultivated in the Mimic Cancer Environment. Microbiol Spectr. 2023 Aug 17;11(4):e00394-23. doi:10.1128/spectrum.00394-23

11. Li J, Zhang C, Huang X, Sun H, Yang L, Liu H, et al. Candida albicans synergizes with Fusobacterium nucleatum in colorectal cancer progression via the Flo9-RadD interaction. Cancer Cell. 2026 May;44(5):1063-1079.e8. doi:10.1016/j.ccell.2026.02.001

12. Zhou T, Solis NV, Marshall M, Yao Q, Pearlman E, Filler SG, et al. Fungal Als proteins hijack host death effector domains to promote inflammasome signaling. Nat Commun. 2025 Feb 12;16(1):1562. doi:10.1038/s41467-025-56657-5

13. Almeida RS, Brunke S, Albrecht A, Thewes S, Laue M, Edwards JE, et al. The Hyphal-Associated Adhesin and Invasin Als3 of Candida albicans Mediates Iron Acquisition from Host Ferritin. PLoS Pathog. 2008 Nov 21;4(11):e1000217. doi:10.1371/journal.ppat.1000217

14. Lin J, Oh SH, Jones R, Garnett JA, Salgado PS, Rusnakova S, et al. The Peptide-binding Cavity Is Essential for Als3-mediated Adhesion of Candida albicans to Human Cells. J Biol Chem. 2014 Jun;289(26):18401–12. doi:10.1074/jbc.M114.547877

15. Lima BP, Shi W, Lux R. Identification and characterization of a novel *Fusobacterium nucleatum* adhesin involved in physical interaction and biofilm formation with *Streptococcus gordonii*. MicrobiologyOpen. 2017 Jun;6(3):e00444. doi:10.1002/mbo3.444

16. Muchova M, Balacco DL, Grant MM, Chapple ILC, Kuehne SA, Hirschfeld J. Fusobacterium nucleatum Subspecies Differ in Biofilm Forming Ability in vitro. Front Oral Health. 2022 Mar 15;3:853618. doi:10.3389/froh.2022.853618

17. Guo P, Tian Z, Kong X, Yang L, Shan X, Dong B, et al. FadA promotes DNA damage and progression of Fusobacterium nucleatum-induced colorectal cancer through up-regulation of chk2. J Exp Clin Cancer Res. 2020 Dec;39(1):202. doi:10.1186/s13046-020-01677-w

18. Rubinstein MR, Wang X, Liu W, Hao Y, Cai G, Han YW. Fusobacterium nucleatum Promotes Colorectal Carcinogenesis by Modulating E-Cadherin/β-Catenin Signaling via its FadA Adhesin. Cell Host Microbe. 2013 Aug;14(2):195–206. doi:10.1016/j.chom.2013.07.012

19. Rubinstein MR, Baik JE, Lagana SM, Han RP, Raab WJ, Sahoo D, et al. Fusobacterium nucleatum promotes colorectal cancer by inducing Wnt/β-catenin modulator Annexin A1. EMBO Rep. 2019 Apr 1;20(4):EMBR201847638. doi:10.15252/embr.201847638

20. Zuraik AA, Daboul Y, Awama MA, Yazigi H, Kayasseh MA, Georges M. Rapid detection of FadA in Fusobacterium nucleatum using the quantitative LAMP colorimetric phenol red method in stool samples from colorectal cancer patients. Sci Rep. 2024 Jun 14;14(1):13739. doi:10.1038/s41598-024-62846-x

21. Purcell RV, Visnovska M, Biggs PJ, Schmeier S, Frizelle FA. Distinct gut microbiome patterns associate with consensus molecular subtypes of colorectal cancer. Sci Rep. 2017;7(1). doi:10.1038/s41598-017-11237-6

22. Yeoh YK, Chen Z, Wong MCS, Hui M, Yu J, Ng SC, et al. Southern Chinese populations harbour non-nucleatum Fusobacteria possessing homologues of the colorectal cancer-associated FadA virulence factor. Gut. 2020 Nov;69(11):1998–2007. doi:10.1136/gutjnl-2019-319635

23. Bista PK, Pillai D, Roy C, Scaria J, Narayanan SK. Comparative Genomic Analysis of Fusobacterium necrophorum Provides Insights into Conserved Virulence Genes. Microbiol Spectr. 2022 Dec 21;10(6):e00297-22. doi:10.1128/spectrum.00297-22

24. Crowley C, Selvaraj A, Hariharan A, Healy CM, Moran GP. Fusobacterium nucleatum subsp. polymorphum recovered from malignant and potentially malignant oral disease exhibit heterogeneity in adhesion phenotypes and adhesin gene copy number, shaped by inter-subspecies horizontal gene transfer and recombination-derived mosaicism. Microb Genomics. 2024 Mar 26;10(3). doi:10.1099/mgen.0.001217

25. Sekizuka T, Ogasawara Y, Ohkusa T, Kuroda M. Characterization of Fusobacterium varium Fv113-g1 isolated from a patient with ulcerative colitis based on complete genome sequence and transcriptome analysis. PLOS ONE. 2017 Dec 7;12(12):e0189319. doi:10.1371/journal.pone.0189319

26. Han YW, Ikegami A, Rajanna C, Kawsar HI, Zhou Y, Li M, et al. Identification and Characterization of a Novel Adhesin Unique to Oral Fusobacteria. J Bacteriol. 2005 Aug;187(15):5330–40. doi:10.1128/JB.187.15.5330-5340.2005

27. Tran HNH, Thu TNH, Nguyen PH, Vo CN, Doan KV, Nguyen Ngoc Minh C, et al. Tumour microbiomes and Fusobacterium genomics in Vietnamese colorectal cancer patients. Npj Biofilms Microbiomes. 2022 Oct 29;8(1):87. doi:10.1038/s41522-022-00351-7

28. Umaña A, Sanders BE, Yoo CC, Casasanta MA, Udayasuryan B, Verbridge SS, et al. Utilizing Whole *Fusobacterium* Genomes To Identify, Correct, and Characterize Potential Virulence Protein Families. J Bacteriol. 2019 Dec;201(23). doi:10.1128/JB.00273-19

29. Abed J, Emgård JEM, Zamir G, Faroja M, Almogy G, Grenov A, et al. Fap2 Mediates Fusobacterium nucleatum Colorectal Adenocarcinoma Enrichment by Binding to Tumor-Expressed Gal-GalNAc. Cell Host Microbe. 2016 Aug;20(2):215–25. doi:10.1016/j.chom.2016.07.006

30. Kaplan CW, Ma X, Paranjpe A, Jewett A, Lux R, Kinder-Haake S, et al. *Fusobacterium nucleatum* Outer Membrane Proteins Fap2 and RadD Induce Cell Death in Human Lymphocytes. Infect Immun. 2010 Nov;78(11):4773–8. doi:10.1128/IAI.00567-10

31. Gur C, Ibrahim Y, Isaacson B, Yamin R, Abed J, Gamliel M, et al. Binding of the Fap2 Protein of Fusobacterium nucleatum to Human Inhibitory Receptor TIGIT Protects Tumors from Immune Cell Attack. Immunity. 2015 Feb;42(2):344–55. doi:10.1016/j.immuni.2015.01.010

32. Kaplan CW, Lux R, Haake SK, Shi W. The *Fusobacterium nucleatum* outer membrane protein RadD is an arginine-inhibitable adhesin required for inter-species adherence and the structured architecture of multispecies biofilm. Mol Microbiol. 2009 Jan;71(1):35–47. doi:10.1111/j.1365-2958.2008.06503.x

33. Guo L, Shokeen B, He X, Shi W, Lux R. *Streptococcus mutans* SpaP binds to RadD of *Fusobacterium nucleatum* ssp. *polymorphum*. Mol Oral Microbiol. 2017 Oct;32(5):355–64. doi:10.1111/omi.12177

34. Engevik MA, Danhof HA, Auchtung J, Endres BT, Ruan W, Bassères E, et al. Fusobacterium nucleatum Adheres to Clostridioides difficile via the RadD Adhesin to Enhance Biofilm Formation in Intestinal Mucus. Gastroenterology. 2021 Mar;160(4):1301-1314.e8. doi:10.1053/j.gastro.2020.11.034

35. Mu W, Jia Y, Chen X, Li H, Wang Z, Cheng B. Intracellular Porphyromonas gingivalis Promotes the Proliferation of Colorectal Cancer Cells via the MAPK/ERK Signaling Pathway. Front Cell Infect Microbiol. 2020 Dec 23;10:584798. doi:10.3389/fcimb.2020.584798

36. Motosugi S, Takahashi N, Mineo S, Sato K, Tsuzuno T, Aoki-Nonaka Y, et al. Enrichment of Porphyromonas gingivalis in colonic mucosa-associated microbiota and its enhanced adhesion to epithelium in colorectal carcinogenesis: Insights from in vivo and clinical studies. PLOS ONE. 2025 Mar 25;20(3):e0320383. doi:10.1371/journal.pone.0320383

37. Handfield M, Mans JJ, Zheng G, Lopez MC, Mao S, Progulske-Fox A, et al. Distinct transcriptional profiles characterize oral epithelium-microbiota interactions. Cell Microbiol. 2005 Jun;7(6):811–23. doi:10.1111/j.1462-5822.2005.00513.x

38. Kazelnik M, Masri R, Caspi M, Wittenstein A, Tsarfaty I, Borenstein E, et al. *Porphyromonas gingivalis* secreted factors drive epithelial–mesenchymal transition (EMT) through gingipains and an H2S-mediated bacterial defense system. Gut Microbes. 2026;18(1):2647532. doi:10.1080/19490976.2026.2647532

39. Long X, Wong CC, Tong L, Chu ESH, Ho Szeto C, Go MYY, et al. Peptostreptococcus anaerobius promotes colorectal carcinogenesis and modulates tumour immunity. Nat Microbiol. 2019 Sep 9;4(12):2319–30. doi:10.1038/s41564-019-0541-3

40. Putative cell wall binding repeat 2 [Peptostreptococcus stomatis DSM 17678]. NCBI Protein; accession EFM63964.1 [Internet]. [cited 2026 Jul 22]. Available from: https://www.ncbi.nlm.nih.gov/protein/EFM63964.1
